# Dissemination of *mcr-1* and β-lactamase genes among *Pseudomonas aeruginosa*: molecular characterization of MDR strains in broiler chicks and dead-in-shell chicks infections

**DOI:** 10.1186/s12941-024-00669-4

**Published:** 2024-01-28

**Authors:** Mona Salem, Gamal Younis, Asmaa Sadat, Nehal Ahmed Talaat Nouh, Dalal Nasser Binjawhar, Mohamed M. Abdel-Daim, Mohamed Elbadawy, Amal Awad

**Affiliations:** 1 Department of Bacteriology, Mycology and Immunology, Faculty of Veterinary Medicine, Mansoura University, Mansoura, 35516 Egypt; 2 Program Medicine, Department of Microbiology, Batterjee Medical College, 21442 Jeddah, Saudi Arabia; 3 Inpatient Pharmacy, Mansoura University Hospitals, Mansoura, 35516 Egypt; 4 Department of Chemistry, College of Science, Princess Nourah Bint Abdulrahman University, P.O. Box 84428, 11671 Riyadh, Saudi Arabia; 5 Department of Pharmaceutical Sciences, Pharmacy Program, Batterjee Medical College, P.O. Box 6231, 21442 Jeddah, Saudi Arabia; 6 Pharmacology Department, Faculty of Veterinary Medicine, Suez Canal University, Ismailia, 41522 Egypt; 7 Department of Pharmacology, Faculty of Veterinary Medicine, Benha University, Moshtohor, Toukh, 13736 Elqaliobiya Egypt; 8 Department of Pathology, College of Veterinary Medicine, University of Georgia, Athens, GA 30602 USA

**Keywords:** *Pseudomonas aeruginosa*, Broiler chicks, Dead in-shell chicks, ESBLs, *Mcr*-1, Antibiotic susceptibility, Virulence, Biofilm

## Abstract

**Objectives:**

*Pseudomonas aeruginosa* (*P. aeruginosa)* is one of the most serious pathogens implicated in antimicrobial resistance, and it has been identified as an ESKAPE along with other extremely significant multidrug resistance pathogens. The present study was carried out to explore prevalence, antibiotic susceptibility phenotypes, virulence-associated genes, integron (*int*1), colistin (*mcr*-1), and β-lactamase resistance' genes (ESBls), as well as biofilm profiling of *P. aeruginosa* isolated from broiler chicks and dead in-shell chicks.

**Design:**

A total of 300 samples from broiler chicks (n = 200) and dead in-shell chicks (n = 100) collected from different farms and hatcheries located at Mansoura, Dakahlia Governorate, Egypt were included in this study. Bacteriological examination was performed by cultivation of the samples on the surface of both Cetrimide and MacConkey’s agar. Presumptive colonies were then subjected to biochemical tests and Polymerase Chain Reaction (PCR) targeting 16S rRNA. The recovered isolates were tested for the presence of three selected virulence-associated genes (*las*B, *tox*A, and *exo*S*).* Furthermore*,* the retrieved isolates were subjected to phenotypic antimicrobial susceptibility testing by Kirby–Bauer disc diffusion method as well as phenotypic detection of ESBLs by both Double Disc Synergy Test (DDST) and the Phenotypic Confirmatory Disc Diffusion Test (PCDDT). *P. aeruginosa* isolates were then tested for the presence of antibiotic resistance genes (ARGs): *int*1, *mcr*-1, and ESBL genes (OXA-10, OXA-2, VEB-1, SHV, TEM, and CTX-M). Additionally, biofilm production was examined by the Tube Adherent method (TA) and Microtiter Plate assay (MTP)**.**

**Results:**

Fifty –five isolates were confirmed to be *P. aeruginosa,* including 35 isolates from broiler chicks and 20 isolates from dead in-shell chicks. The three tested virulence genes (*las*B, *tox*A, and *exo*S*)* were detected in all isolates. Antibiogram results showed complete resistance against penicillin, amoxicillin, ceftriaxone, ceftazidime, streptomycin, erythromycin, spectinomycin, and doxycycline, while a higher sensitivity was observed against meropenem, imipenem, colistin sulfate, ciprofloxacin, and gentamicin. ESBL production was confirmed in 12 (21.8%) and 15 (27.3%) isolates by DDST and PCDDT, respectively. Antibiotic resistance genes (ARGs): *int*1, *mcr*-1, and ESBL genes (OXA-10, SHV, TEM, and CTX-M), were detected in 87.3%, 18.2%, 16.4%, 69.1%, 72.7%, and 54.5% of the examined isolates respectively, whereas no isolate harbored the *OXA*-2 or *VEB*-1 genes. Based on the results of both methods used for detection of biofilm formation, Kappa statistics [kappa 0.324] revealed a poor agreement between both methods.

**Conclusions:**

the emergence of *mcr*-1 and its coexistence with other resistance genes such as β-lactamase genes, particularly *bla*_OXA-10,_ for the first time in *P. aeruginosa* from young broiler chicks and dead in-shell chicks in Egypt pose a risk not only to the poultry industry but also to public health.

**Graphical Abstract:**

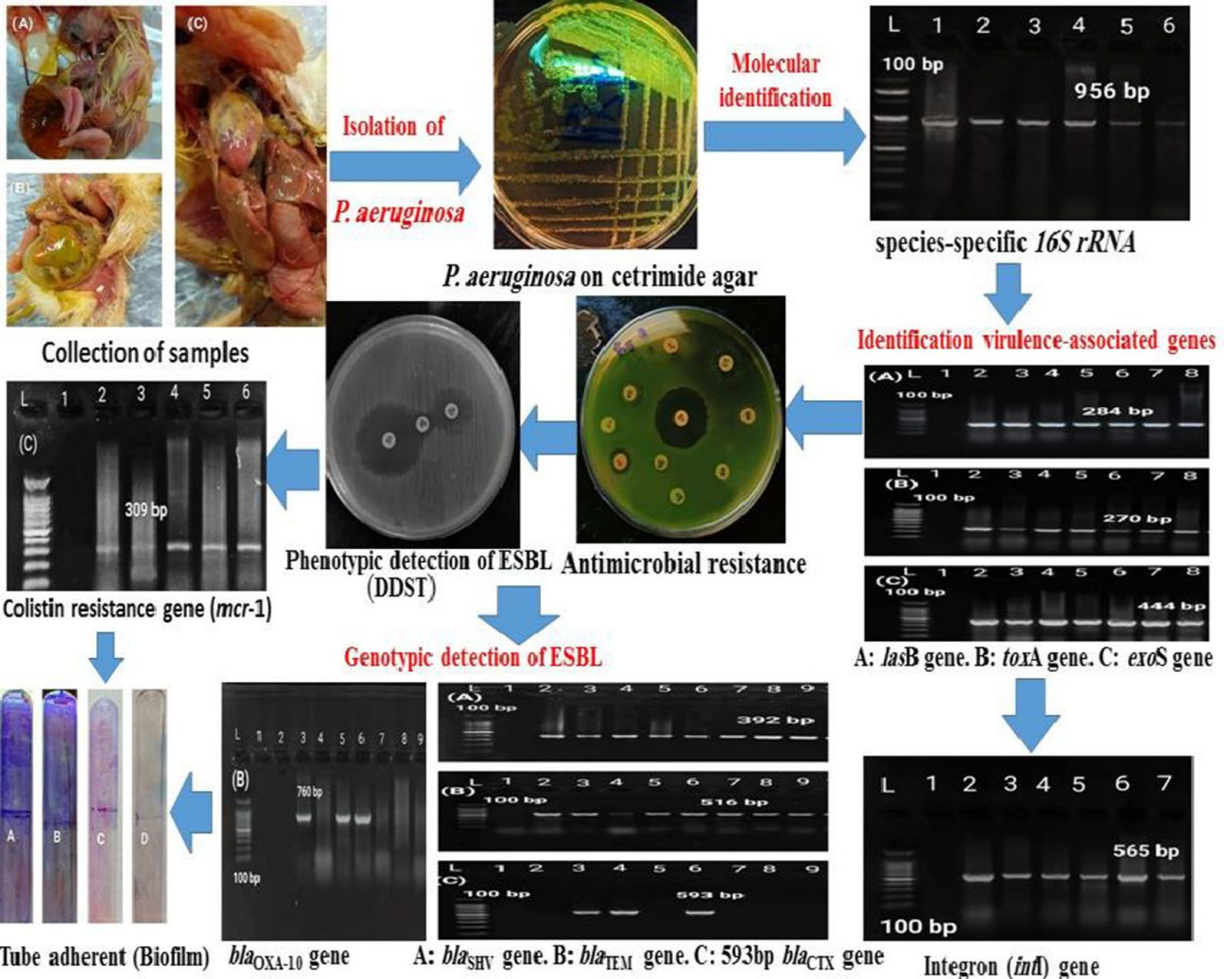

**Supplementary Information:**

The online version contains supplementary material available at 10.1186/s12941-024-00669-4.

## Introduction

*Pseudomonas aeruginosa* is an opportunistic pathogen that normally inhabits humans and avian species. It becomes pathogenic under stress factors such as immunosuppression or the presence of other concomitant infections, causing severe clinical manifestations [1]. *P. aeruginosa* infection in humans is a major threat to public health that is primarily caused by occupational contact with poultry carcasses or related products [2]. It is capable of infecting bird tissues, especially newly hatched chicks, and causing septicemia, respiratory symptoms, diarrhea, cheesy deposits on the air sacs, perihepatitis, pericarditis, and congestion of internal organs [3]. The infection has been related to severe losses in young chicks as well as high embryonic mortality in hatcheries [4].

*P. aeruginosa* exhibits numerous multifactorial virulence factors that contribute to its virulence and pathogenicity. Many virulence factors have been reported such as exotoxins: *exo*A, exoenzymes S, T, and U, exoproteases (alkaline protease, elastase, staphylolysin, and protease IV), pyocyanin, pyoverdine, flagella, and pili [5]. There are two kinds of elastases: LasA (staphylolysin) and LasB (pseudolysin). LasB is a metalloprotease that degrades elastin, the complement system, immunoglobulin A, IgG, mucins, fibrin, collagen, and surfactant proteins A and D. Furthermore, it damages tight junctions in the respiratory tract epithelium by increasing permeability and induces inflammation by increasing IL-8 production [6]. Exotoxin A is the most toxic virulence factor found in *P. aeruginosa*. It suppresses protein synthesis and has a necrotic effect on cells, resulting in cell death and aiding in the invasion and colonization processes [7]. Type 3 Secretion System (T3SS), as exoenzyme S (*exo*S) injects exotoxins or proteins into the cytoplasm of eukaryotic cells and alter their cytoskeletal structure to help in immunity evasion with elevation and increased mortality rates [6].

Antibiotic usage in food-producing animals increased as a result of the shift to large-scale intensive production systems and the increase in consumer demand for chicken products in low- and middle-income countries, especially those in Africa [8]. In Egypt, poultry industry has grown rapidly as a result of local governments' encouragement of it in order to ensure a constant supply of animal products. According to estimates made by Maged et al. [9], 84% of the total meat production came from broilers. Concurringly, a significant reliance on antibiotic use for disease prevention and control has arisen from inadequate biosecurity, poor hygiene, and poor sanitation in poultry production systems in these countries [10]. There have been reports of a higher prevalence of drug-resistant bacteria, notably ESBL-producing bacteria, in chicken production systems in Egypt [11–14].

Markedly, *P. aeruginosa* is one of the most serious pathogens implicated in antimicrobial resistance, and it has been identified as an ESKAPE along with other extremely significant multidrug resistance (MDR) pathogens (non-susceptible to at least one antimicrobial agent in three or more antimicrobial categories) [15]. Antibiotic resistance mechanisms are categorized as intrinsic, acquired, or adaptive. *P. aeruginosa* displays intrinsic resistance to a range of antimicrobials (β-lactam and penem group of antibiotics) because of its outer membrane with low permeability [16]. However, its Intrinsic resistance is also caused by a few other factors such as the expression of efflux pumps, which expel drugs out of cells, as well as the production of antibiotic-inactivating enzymes [17]. Acquired resistance can emerge through mutational shifts or the horizontal transfer of resistance genes by mobile genetic elements (MGEs) such as integrons or plasmids [17]. Class 1 integron (*int*1) is the most prevalent integron observed in clinically isolated *P. aeruginosa* strains [18]. It is a crucial component of integrons, as it harbors genes encoding extended-spectrum β-lactamases, which hydrolyze the third and fourth-generation cephalosporins. Therefore, it is responsible for the introduction of novel antibiotic resistant *P. aeruginosa* strains [19].

Colistin is one of polypeptide antibiotics that has been identified as a critical choice for the treatment of infectious diseases caused by MDR-*P. aeruginosa*. However, the discovery of transferable plasmids conferring colistin resistance genes (*mcr*-1) has created new difficulties in medical science [20]. Colistin resistance has recently emerged in *P. aeruginosa* as a result of its extensive applications in veterinary medicine to control infections and promote growth. Its resistance has become a major concern in the treatment of potentially fatal infections. Particularly, when *mcr*-1 genes coexist with other resistance genes such as ESBL and MBL genes, with the possibility of pan-drug resistance emerging [21]. According to recent studies, *P. aeruginosa* is growing more resistant to colistin, which is considered as global threats to human and veterinary medicine [22].

One of *P. aeruginosa's* most problematic characteristics is its ability to form a biofilm, which increases resistance and virulence and makes it difficult to eradicate [23]. It is a tightly packed population of microorganisms that grow on a range of biotic and abiotic surfaces. Biofilm producing bacteriasurround themselves with an extracellular matrix that serves as a structural scaffold as well as a protective barrier [24]. Bacteria within biofilm stay latent and hidden from the immune system, they may cause local tissue damage and later cause an acute infection. Furthermore, by deactivating the antimicrobial targets or lowering the requirements for the cellular function that the antimicrobials interfere with, bacteria can become more resistant to antimicrobial therapy. As a result, infections linked to biofilms are usually long-lasting infections which develop slowly, are seldom cleared by the immune system, and respond poorly to antibiotics [25]. Biofilm is thought to be the hot spot for the accumulation and transmission of antibiotic resistance genes (ARGs) and also acts as a source of infection [26]. Therefore, this study was carried out to explore the prevalence, virulence-associated genes, antibiotic susceptibility phenotypes, as well as some genetic elements associated with antimicrobial resistance, including integron (*int*1), colistin (*mcr*-1), β-lactamase resistant (ESBls) genes, and biofilm profiling of *P. aeruginosa* isolates obtained from broiler chicks and dead in-shell chicks.

## Materials and methods

### Ethical approval

This study was ethically approved by the Research Ethics Committee of the Faculty of Veterinary Medicine, Mansoura University, Egypt (Protocol code: M/104).

### Sample collection

**Fig. 1:**
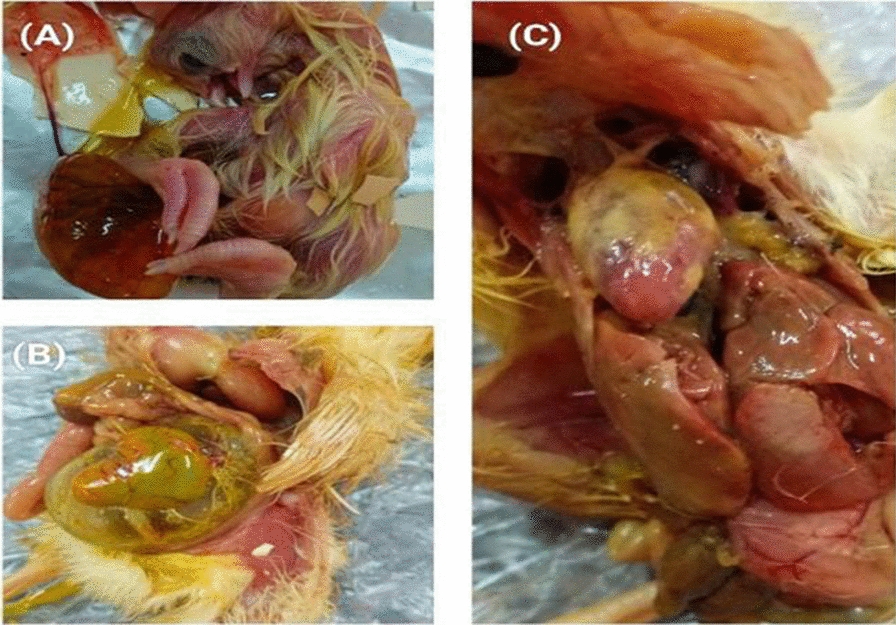
The most common lesions detected during post-mortem examination. **A**, **B** Unabsorbed yolk sac in dead in-shell chicks and 1-day old chick. **C** Newly hatched chicks with congested internal organs, fibrinous pericarditis, and perihepatitis

A total of 300 samples were randomly collected from different hatcheries and farms located atat Mansoura, Dakahlia Governorate, Egypt. Out of them, 100 samples of yolk sacs from dead in-shell chicks and 200 samples from diseased and freshly dead broiler chicks (1–15 days old) including unabsorbed yolk sac, internal organs such as liver, lungs, and heart (three organs per each bird) in the period between March 2021 to November 2021. Most of these cases showed diarrhea, ruffled feathers, respiratory manifestations, and signs of septicemia (petechial hemorrhages on the internal organs and serous membranes, swollen and hyperemic liver and spleen, swollen and congested kidneys). The most common lesions detected during post-mortem examination were pericarditis, air sacculitis, perihepatitis, and omphalitis (Fig. 1). The cervical dislocation was used to euthanize diseased chicks in a humane manner [27]. Under strict sanitary conditions, samples were collected in a closed, labeled sterile containers to avoid cross-contamination according to the WOAH protocol for sample collection. Sterilization of egg samples was done by cleaning the exterior shell with 70% alcohol, cricking with a sterile knife, and emptying the contents into sterile bags. All samples were immediately transported in sterile ice containers to the Bacteriology, Mycology, and Immunology Department, Faculty of Veterinary Medicine, Mansoura University, Egypt, for bacteriological examination.

### Isolation and identification of *P. aeruginosa*

A bacteriological examination was performed following Shukla and Mishra [28]. Under strict aseptic conditions, visceral organ samples (liver, heart, lung, and unabsorbed yolk sac) were collected from diseased and freshly deceased broiler chicks, as well as the yolk sac of dead in-shell chicks. All samples were pre-enriched in 9 mL buffer peptone water (PBW) (Oxoid Ltd, Hampshire, England) and incubated aerobically at 37 °C for 24 h. A loopful of inoculated broth was streaked onto the surface of Cetrimide and MacConkey’s agar (Oxoid Ltd, Hampshire, England) and incubated aerobically for 24–48 h at 37 °C. Suspected colonies appeared as large, transparent (non-lactose fermenter) on MacConkey’s agar, and yellowish green pigmentation with a fruity smell on Cetrimide agar were selected and purified on Trypticase Soya Agar (TSA) (Oxoid Ltd, Hampshire, England). Purified colonies were examined morphologically by gram’s staining and characterized biochemically using catalase, oxidase, indole, methyl red, voges-proskauer, citrate utilization, nitrate reduction, and glucose fermentation tests [29]. For the further identification, all suspected strains were preserved in 70% glycerol at − 20 °C.

### Molecular identification of *P. aeruginosa*

DNA was extracted from biochemically suspected *P. aeruginosa* isolates using the boiling method [30]. In general, three to five suspected colonies were selected, inoculated in 3 mL of tryptic soy broth, and then cultured for 18 h at 37 °C. One milliliter from the overnight bacterial suspension was centrifuged at 8000*g* for 2 min and the supernatant was discarded. The bacterial pellet was resuspended in 1 ml nuclease-free water and heated at 95 °C for 20 min using a thermal block (Biometra). The supernatant was used as a DNA template and stored at − 20 °C for further molecular characterization. About 1 μL from each extracted DNA was used to determine the concentration and purity of DNA samples using a NanoDrop 1000 spectrophotometer (Thermo Fisher) at 260 nm.

For confirmation of *P. aeruginosa,* a PCR assay using a species-specific primer targeted 16S rRNA (Metabion, Germany) was used according to Spilker et al. [31]. The primer sequences, amplicon size, and PCR condition are illustrated in Table 1. The PCR products were separated by gel electrophoresis (Hoefer PS 300-B li-fe science, USA) in 1% agarose gel stained with ethidium bromide in 1 × TBE buffer at 0.8% (w/v). The fragment sizes were determined by using a 100 bp DNA ladder (Qiagen, USA). A negative control (nuclease-free water) was used. For gel analysis, 10 μL of the products were loaded in each gel lane, then the gel was visualized by a UV transilluminator system (cleaver scientific ltd UV gel documentation system, Rugby, Warwickshire, UK).

**Table 1:** Oligonucleotides used in this study

Target gene	Primer direction and sequence	Amplicon size (bp)	References
*16S* rRNA	F: GGGGGATCTTCGGACCTCA R: TCCTTAGAGTGCCCACCCG	956	[31]
*las*B	F: GGAATGAACGAAGCGTTCTCCGAC R: TTGGCGTCGACGAACACCTCG	284	[33]
*tox*A	F: CTGCGCGGGTCTATGTGCC R: GATGCTGGACGGGTCGAG	270	[33]
*exo*S	F: CGTCGTGTTCAAGCAGATGGTGCTG R: CCGAACCGCTTCACCAGGC	444	[33]
*int1*	F: GCCTTGCTGTTCTTCTACGG R: GATGCCTGCTTGTTCTACGG	565	[34]
*bla*_TEM_	F: ATCAGCAATAAACCAGC R: CCCCGAAGAACGTTTTC	516	[35]
*bla*_SHV_	F: AGGATTGACTGCCTTTTTG R: ATTTGCTGATTTCGCTCG	392	[35]
*bla*_CTX-M_	F: ATG TGC AGY ACC AGT AAR GTK ATG GC R: TGG GTR AAR TAR GTS ACC AGA AYC AGC GG	593	[36]
*bla*_OXA-10_	F:TATCGCGTGTCTTTCGAGTA R: TTAGCCACCAATGATGCCC	760	[37]
*bla*_VEB-1_	F: CGACTTCCATTTCCCGATGC R: GGACTCTGCAACAAATACGC	642	[37]
*bla*_OXA-2_	F: GCCAAAGGCACGATAGTTGT R: GCGTCCGAGTTGACTGCCGG	700	[38]
mcr-1	F: CGGTCAGTCCGTTTGTTC R:CTTGGTCGGTCTGTA GGG	309	[39]

For sequencing of 16S rRNA, the amplified products were extracted from the gel by QIAquick Gel Extraction Kits (Qiagen, USA). All purification procedures were carried out in accordance with the manufacturer guidelines. The purified products were sent for sequencing to Macrogene Company, South Korea. The 16S rRNA sequences were aligned using Bioedit software and compared with sequences available in the National Center for Biotechnology Information (NCBI) database by BLAST search (https://blast.ncbi.nlm.nih.gov/Blast.cgi?PAGE_TYPE=BlastSearch). The neighbor-joining approach was used to create the phylogenetic tree using Mega 11 software [32]. The 16S rRNA gene sequence has been submitted in the gene bank under the accession number: OP481895.

### Detection of *P. aeruginosa* virulence-associated genes

Three virulence-associated genes, including elastase enzyme (*las*B), exotoxin A (*tox*A), and exoenzyme S (*exo*S), were selected and subjected to PCR for their detection using three pairs of primers (Metabion, Germany) [33]. The primer sequences, amplicon size, and PCR condition are mentioned in Table 1.

### Antimicrobial susceptibility testing

In-vitro susceptibility testing of *P. aeruginosa* isolates was conducted by disc-diffusion test according to Koneman et al. [40] using the Kirby–Bauer disc diffusion method [41] against 19 antibiotics discs (Oxoid Ltd, Hampshire, England) comprising 10 different antimicrobial classes of both human and veterinary importance. In brief, the culture of an isolate with 0.5 McFarland density was distributed onto the surface of Müller-Hinton agar (Oxoid Ltd, Hampshire, England). Antimicrobial discs including, penicillin (P, 10 iu), amoxicillin (AX, 10 μg), amoxicillin/clavulanic acid (AMC, 30 μg), imipenem (IPM, 10 μg), meropenem (MEM, 10 μg), ceftriaxone(CRO, 30 μg), cefuroxime (CXM, 30 μg), cefotaxime (CTX, 30 μg), ceftazidime (CAZ, 30 μg), gentamicin(CN, 10 μg), streptomycin (S, 10 μg), amikacin (AK, 30 μg), kanamycin (K, 30 μg), apramycin (APR, 15 μg), colistin (CT, 10 μg), erythromycin (E, 15 μg), spectinomycin (SPT, 100 μg), ciprofloxacin (CIP, 5 μg), and doxycycline (DO, 30 μg) were applied on the agar surfaces. The inoculated plates were incubated at 37 °C for 24 h, and the zones of inhibition were measured and interpreted according to the Clinical and Laboratory Standard Institute recommendations [42] (Additional file 1: Table S1). MDR was defined as non-susceptible to at least one antimicrobial agent in three or more antimicrobial categories and extensively drug resistant (XDR) was defined as non- susceptibility to at least one agent in all but two or fewer antimicrobial categories [43]. MDR index (MDRI) was measured by dividing the numbers of antibiotics to which they were resistant by the total number of antibiotics to which they had been exposed [44].

### Detection of colistin-resistant by the broth microdilution method (BMD)

The BMD method was used for determining the MICs of *P. aeruginosa* isolates, and the results were interpreted following the European Committee on Antimicrobial Susceptibility Testing (EUCAST) breakpoints (susceptible ≤ 2, resistant > 2 mg/L) [45] In brief, lyophilized powder of colistin sulfate salt (Sigma, USA) was reconstituted in distilled water, and a varied required concentration (0.25–16 µg/mL) was created by two fold serial dilutions in separate tubes containing cation-adjusted Mueller–Hinton broth (CAMHB) (Oxoid Ltd, Hampshire, England). *P. aeruginosa* colonies were picked from an 18-h TSA culture and transferred to a CAMHB tube. After incubating the broth overnight at 37 °C, the turbidity was adjusted to a 0.5 McFarland standard, and the suspension was diluted in broth to achieve a final bacterial concentration of 5 × 105 CFU/mL. To achieve a total volume of 100 µL volume in each well of the 96-well U-bottom polystyrene plate A total of 25 µL of drug, 25 µL of bacterium inoculum, and 50 µL of cation-adjusted Mueller Hinton (CAMH) broth were added, and the mixture was incubated at 37 °C for 20 h. The minimum inhibitory concentration (MIC) was defined as the lowest medication concentration that totally ceased visible growth [46]. Phenotypically resistant isolates were then subjected for PCR for detection of *mcr-1* gene and the amplified PCR products were then sent for sequencing.

### Detection of ESBL-producing isolates

#### Double disc synergy test (DDST)

ESBL production for *P. aeruginosa* isolates was detected phenotypically by a double disc synergy test (DDST), as described by Jarlier et al. [47]. A suspension of each isolate to 0.5 McFarland density was distributed onto the Müller-Hinton agar (Oxoid Ltd, Hampshire, England) using a sterile cotton swab. The AMC (20 μg amoxicillin and 10 μg clavulanic acid) disc was placed in the center of the plate, and two discs of 3rd generation cephalosporins (ceftazidime-CAZ 30 μg and cefotaxime-CTX 30 μg) were placed at a 15 mm distance (center to center) from the amoxicillin-clavulanic acid disc. The plates were then incubated overnight at 37 °C. Enhancement of the zone of inhibition of any one of the two drug discs towards amoxicillin-clavulanic acid (D-shape or keyhole shape) was interpreted as synergy indicating an ESBL-positive strain.

#### Phenotypic confirmatory disc diffusion test (PCDDT)

In this test, an overnight culture of each isolate was adjusted to a 0.5 McFarland density suspension and swabbed onto a Müller-Hinton agar plate (Oxoid Ltd, Hampshire, England)) using a sterile cotton swab. The ceftazidime (30 μg) and amoxicillin-clavulanic acid (20 μg/10 μg) discs were placed at a distance of 20 mm apart on the agar plates and incubated overnight at 37 °C. A strain producing a diameter of the inhibition zone of ≥ 5 mm around the disc of amoxicillin-clavulanic acid and not around the disc with ceftazidime alone was considered positive for ESBL [48].

### Detection of genetic elements associated with antimicrobial resistance

Knowing the molecular mechanisms employed by bacteria to resist the effects of antimicrobials is essential in order to identify global developments of resistance, enhance the administration of existing medications, and develop novel approaches to fight resistance in addition to new drugs that are less likely to develop resistance. Uniplex PCR was performed for the detection of six β-lactamase resistance-encoding genes, including class A ESBLs (*bla*_TEM_*, bla*_SHV_ [35], *bla*_CTX-M_ [36], *bla*_VEB_ [37], class D β-lactamases (*bla*_OXA2,_ [38] *bla*_OXA10_ [37] and *int*1 [34] gene. In addition, the *mcr*-1 [39] gene were also amplified from phenotypically resistant isolates. The primer sequences, and amplified segment sizes are all mentioned in Table 1. The amplified products of the *mcr*-1, *int*1, and *bla*_*OXA-10*_ genes were sequenced as previously mentioned and the genes sequence has been submitted in the gene bank under the accession number: OQ077571, OQ077572, and OQ077570, respectively.

### Biofilm formation assay for *P. aeruginosa* isolates

#### Qualitative detection by the tube adherent method (TA)

The tube method was applied to evaluate the biofilm-forming ability of *P. aeruginosa* isolates. A single pure colony was obtained from TSA culture plates and inoculated in a tube that contained 10 mL of tryptic soya broth enhanced with 1% glucose (TSBG) (Oxoid Ltd, Hampshire, England), and then incubated overnight at 37 °C without shaking. A negative control with just TSBG and no bacteria was included as well. Each tube was thoroughly aspirated after incubation, and the adhering biofilm was rinsed using phosphate-buffered saline (PBS, pH 7.4). The tubes were turned upside-down for 30 min until completely dry, then stained with 1% crystal violet for 15 min. The excess dye was rinsed away with phosphate-buffered saline, and the development of biofilms was observed with the naked eye by two different observers [49]. Positive results were seen as a violet color on the tube walls and bottom surfaces. Depending on the intensity of the violet color, *P. aeruginosa* isolates were categorized as strongly adherent, moderately adherent, weakly adherent, or non-adherent. Each isolate was tested in triplicate [49].

##### Quantitative detection by microtiter plate assay (MTP)

The MTP was performed in a 96-well polystyrene microtiter plate with a flat bottom and a lid. A freshly prepared 20 μL bacterial suspension was inoculated into 180 μL of Mueller–Hinton broth with 1% glucose. The mixtures were aliquoted into the microtiter plate and incubated aerobically for 24 h at 37 °C without agitation. A negative control sterile broth was used. After incubation, the cultures were carefully aspirated, and the adhering biofilms were washed three times with 200 μL of PBS (pH 7.4) to eliminate any non-adherent cells. The adherent cells were fixed using 150 μL absolute methanol for 15 min. Methanol was aspirated, and the adhering biofilms were dyed for 20 min with 150 μL of 1% crystal violet at room temperature. The plate was gently washed three times with distilled water and left inverted in the air until dry. The stained biofilm was dissolved with 150 μL of 95% ethanol to detach the fixed cell from the well. The optical density of each well was measured using an ELISA plate reader (Robonic, India) at 560 nm [50]. The interpretation of the obtained results was measured by the definition of the cut-off value that distinguishes biofilm production from non-biofilm-producing strains [51]. A predefined value of 0.1 was adjusted for the optical density of the negative control (ODc). Depending on the optical density of each sample (ODi) and the negative control (ODc), the isolates have been categorized as: strong (4 × ODc < ODi), moderate (2 × ODc < ODi ≤ 4 × ODc), weak (ODc < ODi ≤ 2 × ODc), or non-producers of biofilm (ODi < ODc). Each strain was tested for biofilm production in triplicate [51].

#### Statistical analysis

Statistical package for social sciences software (SPSS Inc. No. 23, Version 23.0. Armonk, NY: IBM Corp.) was used. The Kappa test was used to find agreement between TA and MTP for the detection of biofilm formation. A Kappa value of < 0.4 is considered poor agreement, from 0.4 to 0.75 is considered moderate to good, and value of > 0.75 represents excellent agreement. A Kappa value of 1.0 means that there is perfect agreement between all raters. If the P-value > 0.05, the result is not statistically significant, while if the P-value < 0.05, the result is statistically significant, and P-value < 0.01, the result is highly statistically significant.

## Results

A total of 300 samples from broiler chicks (n = 200) and dead in-shell chicks (n = 100) were collected in this study to isolate *P. aeruginosa,* the collected samples were cultivated on the surface of Cetrimide and MacConkey’s agar. Presumptive isolates were identified biochemically and by PCR targeting 16S rRNA. Furthermore, *P. aeruginosa* were tested for the presence of three selected virulence-associated genes (*las*B, *tox*A, and *exo*S) and antimicrobial susceptibility testing by disc diffusion method, phenotypic detection of ESBLs, and gentotypic detection of  *int*1, *mcr*-1, and ESBL genes (OXA-10,OXA-2, VEB-1, SHV, TEM, and CTX-M). Additionally, biofilm production was examined by the TA and MTP.

### Phenotypic characteristics of the recovered *P. aeruginosa* isolates

In this investigation, the results of bacteriological examination based on cultural and morphological characteristics yielded 80 (80/300; 26.6%) suspected *P. aeruginosa* isolates. These isolates were stained negative by gram staining, produced bluish-green pigment with a fruity grape-like odor on cetrimide agar, and had a pale colony with no lactose fermentation on Macconkey's agar plates**.** Biochemically, the retrieved isolates were positive for catalase, citrate, methyl-red, urease, and oxidase tests and negative for both indole and voges proskauer tests.

### Prevalence molecular identification of *P. aeruginosa*

**Fig. 2:**
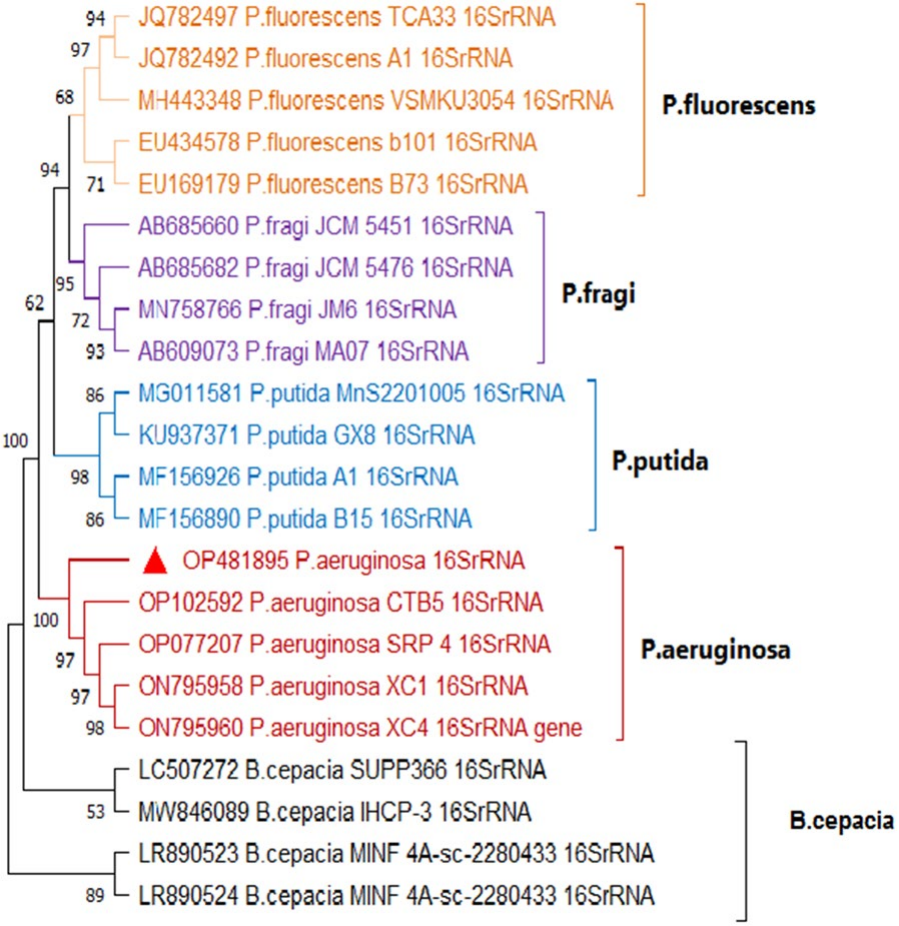
Phylogenetic tree showing the genetic relatedness among *Pseudomonas spp.* based on nucleotide sequence analysis of the 16S rRNA gene. Strain in this study is labeled with a red triangle. DNA sequence analysis of the 16S rRNA gene of the *P. aeruginosa* strain showed high similarity with other *Pseudomonas spp*. from different sources and countries

Using a PCR assay 16S rRNA was identified (Additional file 1: Fig. S1), *P. aeruginosa* was successfully confirmed in 55 isolates with an overall prevalence of 18.3% (55/300), including 20 isolates from dead in-shell chicks (20%, 20/100) and 35 isolates from diseased and freshly dead broiler chicks (17.5%, 35/200). One representative isolate's purified product was sequenced and submitted to the GenBank database under the accession number OP481895. DNA sequence analysis of the 16S rRNA gene of the *P. aeruginosa* strain showed a similarity percentage ranging from 80 to 97% with other *Pseudomonas spp*. from different sources and countries (Fig. 2).

### Distribution of virulence-associated genes

**Fig. 3:**
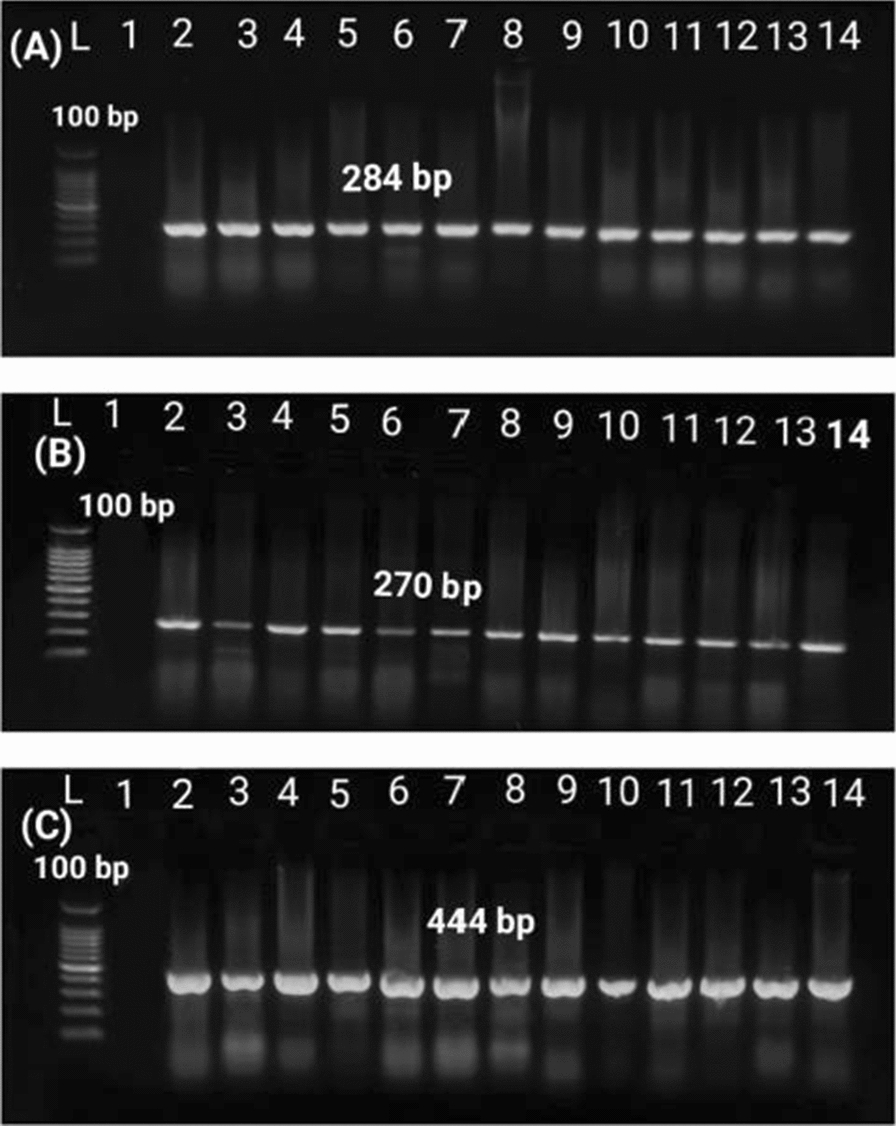
Agarose gel electrophoresis showing amplification of virulence-associated genes. **A** 284 bp fragment of *las*B gene. **B** 270 bp fragment of *tox*A gene. **C** 444 bp fragment of *exo*S gene of *P. aeruginosa* isolates. L: 100 bp ladder. 1: Control negative

*P. aeruginosa* isolates were screened for the presence of three selected virulence gene markers by PCR, and the results revealed that *tox*A, *las*B, and *exo*S genes were successfully amplified from all *P. aeruginosa* isolates (Fig. 3; Table 2).

**Table 2:** Distribution of virulence-associates genes and antimicrobial-resistance ‘genes in *P. aeruginosa* isolates

Genes	*P. aeruginosa* isolates no. (%)
*las*B	55 (100%)
*tox*A	55 (100%)
*exo*S	55 (100%)
*int*1	48 (87.3%)
*mcr*-1	16 (29.1%)
*bla*_TEM_	40 (72.7%)
*bla*_SHV_	38 (69.1%)
*bla*_CTX-M_	30 (54%)
*bla*_OXA-10_	9 (16.4%)
*bla*_VEB-1_	0 (0.00%)
*bla*_OXA-2_	0 (0.00%)

### Antimicrobial susceptibility of *P. aeruginosa* isolates

**Fig. 4:**
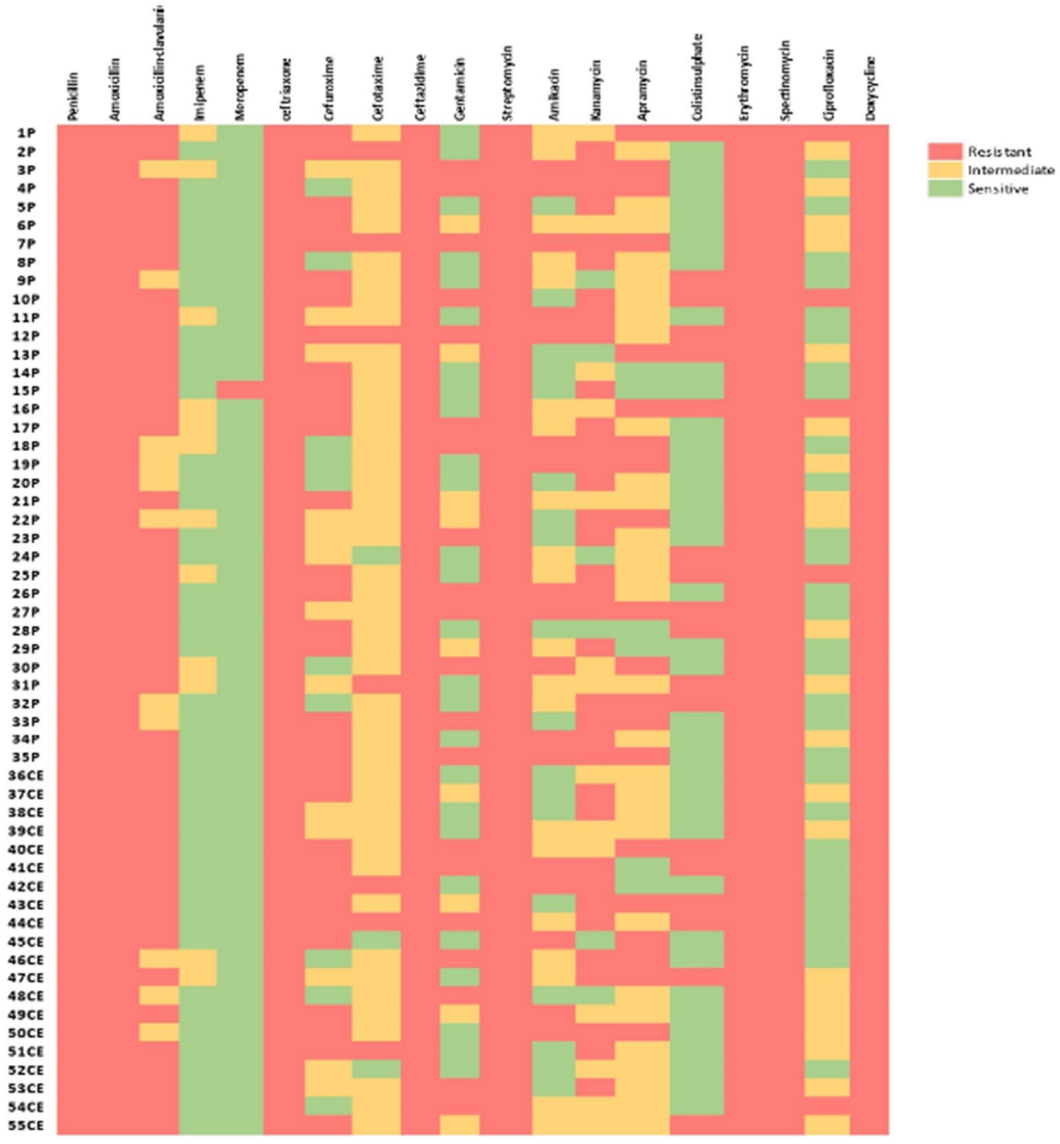
A heat map showing the results of antimicrobial susceptibility testing. All isolates were completely resistant to penicillin, amoxicillin, ceftriaxone, ceftazidime, streptomycin, erythromycin, spectinomycin, and doxycycline (100% each). A high resistance was observed against amoxicillin-clavulanic, kanamycin, and cefuroxime. While 100% of *P. aeruginosa* isolates were completely sensitive to meropenem, and high sensitivity was observed to imipenem, colistin sulfate, ciprofloxacin, and gentamicin

The results of antimicrobial susceptibility testing revealed that all isolates were completely resistant to penicillin, amoxicillin, ceftriaxone, ceftazidime, streptomycin, erythromycin, spectinomycin, and doxycycline (100% each). As well, high resistance was observed against amoxicillin-clavulanic (80%), kanamycin (63.6%), and cefuroxime (58.2%). An intermediate resistance was observed against cefotaxime (81.8%), apramycin (52.7%), and amikacin (36.4%), while all *P. aeruginosa* isolates were completely sensitive to meropenem (100%), and high sensitivity was observed to imipenem (78.2%), colistin sulfate (67.3%), ciprofloxacin (52.7%), and gentamicin (47.3%) (Table 3; Fig. 4). Interestingly, all *P. aeruginosa* isolates were MDR, as the isolates were resistant to at least one member of six different tested classes. The most prevalent antimicrobial resistance profiles were P, AX, CRO, CAZ, S, E, SPT, DO, AMC, K, and CXM (Additional file 1: Table S2). Interestingly, extensive drug resistance (XDR) was noticed among two isolates that were resistant to 15 types of the tested antibiotics. The overall MDRI ranged from 0.5 to 0.8 (Additional file 1: Table S1). A total of 10 (18.2%) isolates had MICs above the EUCAST breakpoints (resistant > 2 mg/L) and were phenotypically considered as colistin resistant isolates.

**Table 3:** Antimicrobial susceptibility testing results by Kirby–Bauer disc diffusion method

Antimicrobial agent	Family	Disc code	CPD (μg)	*P. aeruginosa*					
				Resistant		Intermediate		Sensitive	
				No.	%	No.	%	No.	%
Penicillin	β-lactams	P	10	55	100	0	0.00	0	0.00
Amoxicillin		AX	10	55	100	0	0.00	0	0.00
Amoxicillin-clavulanic	Penicillin-like, beta lactamase inhibitor	AMC	30	44	80	11	20	0	0.00
Imipenem	Carbapenems	IPM	10	0	0.00	12	21.8	43	78.2
Meropenem		MEM	10	0	0.00	0	0.00	55	100
Ceftriaxone	Cephalosporin	CRO	30	55	100	0	0.00	0	0.00
Cefuroxime		CXM	30	32	58.2	13	23.6	10	18.2
Cefotaxime		CTX	30	7	12.7	45	81.8	3	5.5
Ceftazidime		CAZ	30	55	100	0	0.00	0	0.00
Gentamicin	Aminoglycoside	CN	10	20	36.4	9	16.4	26	47.3
Streptomycin		S	10	55	100	0	0.00	0	0.00
Amikacin		AK	30	17	30.9	20	36.4	18	32.7
Kanamycin		K	30	35	63.6	14	25.5	6	10.9
Apramycin		APR	15	20	36.4	29	52.7	6	10.9
Colistin sulphate	Polypeptides	CT	10	18	32.7	0	0.00	37	67.3
Erythromycin	Macrolides	E	15	55	100	0	0.00	0	0.00
Spectinomycin	Aminocyclitol	SPT	100	55	100	0	0.00	0	0.00
Ciprofloxacin	Fluoroquinolone	CIP	5	5	9	21	38.2	29	52.7
Doxycycline	Tetracycline	DO	30	55	100	0	0.00	0	0.00

### Drug resistant of *P. aeruginosa isolates* according to the public health hazard.

By studying the antimicrobial resistance of *P. aeruginosa* isolates recovered from broiler chicks and dead in-shell chicks concerning their importance to human health (Table 4), all isolates showed complete resistance towards penicillin, amoxicillin, ceftriaxone, ceftazidime, streptomycin, and erythromycin (100% each), followed by amoxicillin-clavulanic, kanamycin, cefuroxime, gentamicin, amikacin, apramycin, and colistin sulfate (80%, 63.6%, 58.2%, 36.4%, 30.9%, 36.4%, and 32.7%, respectively), which are all classified according to the World Health Organization [52] as a critical antibiotic for human consumption (Category I). Furthermore, complete resistance to highly important antibiotics was observed: doxycycline (100%) (Category II). Moreover, all isolates exhibited complete resistance to spectinomycin (100%), which was identified as an important medicine for human use (Category III).

**Table 4:** Classification of antibiotics categories based on their importance in human and veterinary medicine according to World Health Organization (WHO)

Antibiotic	Disc conc	Antimicrobial group	Antimicrobial category	Medical importance
Penicillin Amoxicillin (AX)	10 IU 10 μg	β-lactams	I	Critically important
Amoxicillin-clavulanic (AMC)	30 μg	Penicillin-like, -beta-lactamase inhibitor	I	Critically important
Imipenem (IPM) Meropenem (MEM)	10 μg 10 μg	Carbapenems	I	Critically important
Ceftriaxone (CRO) Cefuroxime (CXM) Cefotaxime (CTX) Ceftazidime (CAZ)	30 μg 30 μg 30 μg 30 μg	Cephalosporin 3rd generation	I	Critically important
Gentamicin (CN) Streptomycin (S) Amikacin (AK) Kanamycin (K) Apramycin (APR)	10 μg 10 μg 30 μg 30 μg 15 μg	Aminoglycoside	I	Critically important
Colistin sulfate (CT)	10 μg	Polypeptides	I	Critically important
Erythromycin (E)	15 μg	Macrolides	I	Critically important
Ciprofloxacin (CIP)	5 μg	Fluoroquinolone	I	Critically important
Doxycycline (DO)	30 μg	Tetracycline	II	Highly Important
Spectinomycin (SPT)	100 μg	Aminocyclitol	III	Important

### Phenotypic and genotypic detection of ESBL-producing isolates

**Fig. 5:**
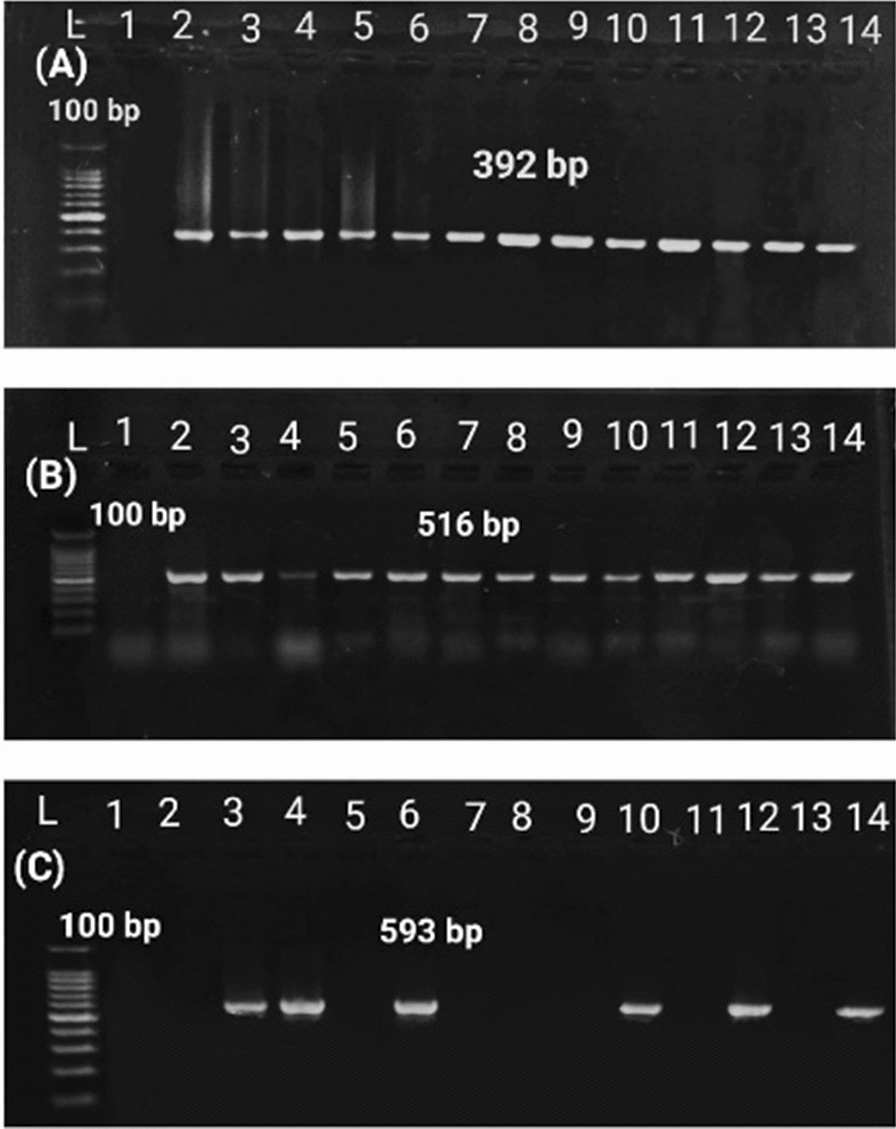
Agarose gel electrophoresis showing amplification of β-lactamase genes*.* **A** 392 bp fragment of *bla*_SHV_ gene. **B** 516 bp fragment of *bla*_TEM_ gene. **C** 593 bp fragment of gene *bla*_CTX-M_. L: 100 bp ladder. 1: Control negative

Out of 55 isolates tested for ESBL production by DDST, 12 (21.8%) isolates were confirmed as ESBL producers. Positive isolates showed enhancement in the zone of inhibition in one or two tested discs (cefotaxime-CTX 30 μg, ceftazidime-CAZ 30 μg) towards the amoxicillin-clavulanic acid disc (D-shape or keyhole shape). While, PCDDT identify 15 (27.3%) ESBL producer isolates. The isolates exhibited a significant enhancement in the zone size with the combination disc amoxicillin-clavulanic acid (≥ 5 mm) when compared with ceftazidime discs alone. The PCDDT detected the same 12 strains of DDST as well as an additional 3 strains as ESBL producers. Genotypically, the distribution of *bla*_TEM,_ *bla*_SHV,_ *bla*_CTX-M,_ and *bla*_OXA-10_ genes was detected in 40 (72.7%), 38 (69.1%), 30 (54%), and 9 (16.4%) isolates, respectively, while no isolate carried either *bla*_VEB-1_ or *bla*_OXA-2_ genes (Fig. 5).

### Detection of integron (*int*I) gene

**Fig. 6:**
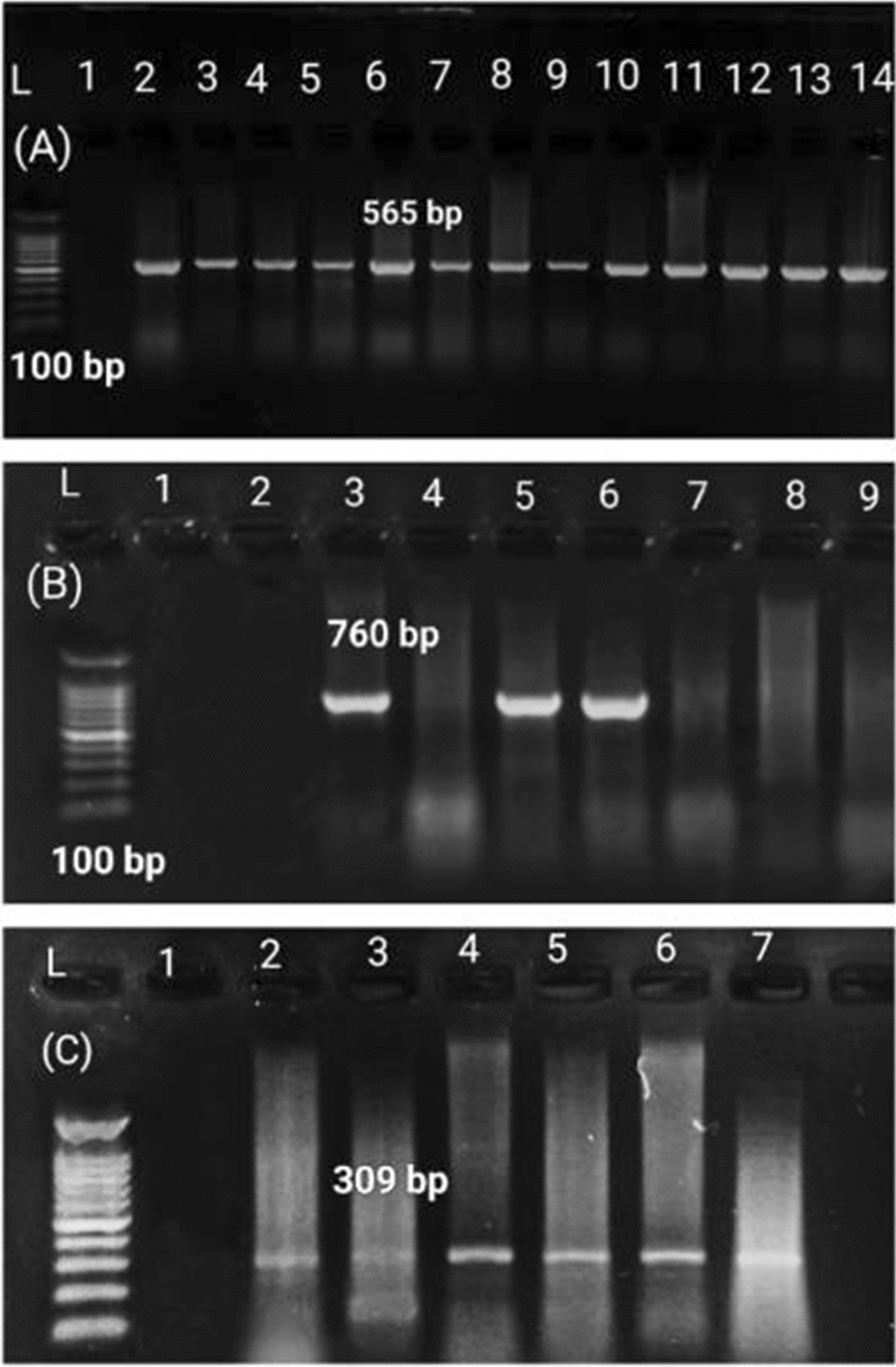
Agarose gel electrophoresis showing amplification of *int*1, *bla*_OXA-10_, and *mcr-*1 genes. **A** 565 bp fragment of *int*1 gene. **B** 760 bp fragment of *bla*OXA-10 gene. **C** 309 bp fragment of *mcr*-1 gene. L: 100 bp ladder 1: Control negative

**Fig. 7:**
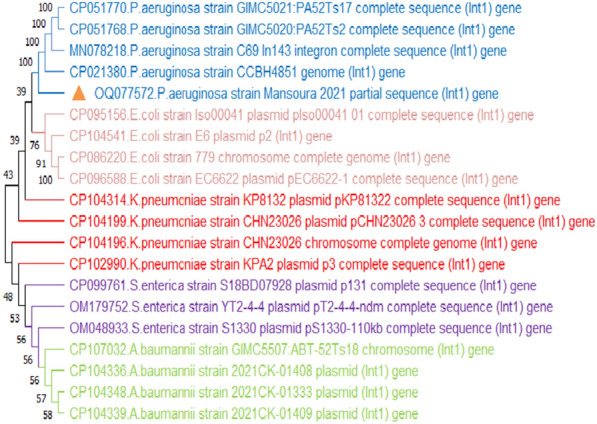
Phylogenetic tree of integron (*Int*1) gene showing 100% homology to *P. aeruginosa int*1 gene and a lower similarity with *Escherichia coli, Klebsiella pneumonia, Salmonella enterica, Acinetobacter baumannii* strains. Strain presented in this study is labeled with a triangle

The PCR assay identified the *int*1 gene in 87.3% (48/55) of the tested *P. aeruginosa* isolates (Fig. 6). A purified product of the amplified *int*1 gene was sequenced and submitted to the GenBank database under the following accession number: OQ077572. The constructed phylogenetic tree showed 100% homology to *P. aeruginosa* (*int*1) gene from the gene bank, while a low similarity was displayed with *Escherichia coli, Klebsiella pneumonia, Salmonella enterica,* and *Acinetobacter baumannii* strains, which were located in separate clades (Fig. 7).

### Detection of colistin resistance gene (*mcr*-1)

**Fig. 8:**
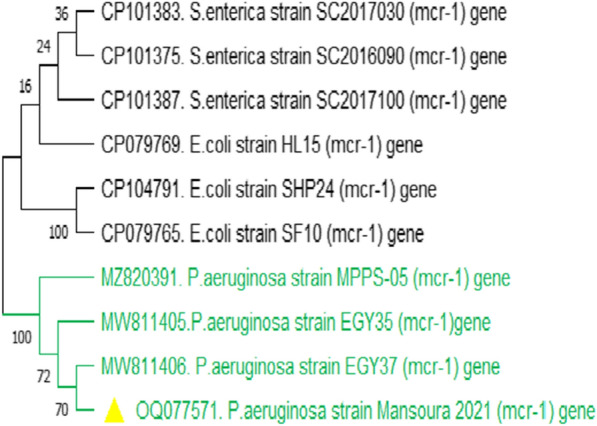
Phylogenetic tree of colistin (*mcr-1*) gene showing high similarity to *P. aeruginosa mcr*-1 gene including two Egyptian strains (MW811405, MW811406) isolated from Bovine Milk. Strain presented in this study is labeled with a yellow triangle

The *mcr*-1 gene was identified by PCR in 18.2% (10/55) of *P. aeruginosa* isolates (Fig. 6). These isolates were resistance to colistin in the Broth Microdilution method (BMD). To our knowledge, this is the first study in Egypt that has reported the detection of the *mcr*-1 gene in *P. aeruginosa* clinical isolates obtained from broiler chicks and dead in-shell chicks. The phylogenetic tree constructed based on the partial sequencing of the *mcr*-1 gene (OQ077571) showed high similarity to the *P. aeruginosa (mcr*-1) gene, including two Egyptian strains (MW811405, MW811406) isolated from bovine milk (Fig. 8).

### Biofilm production capability

**Fig. 9:**
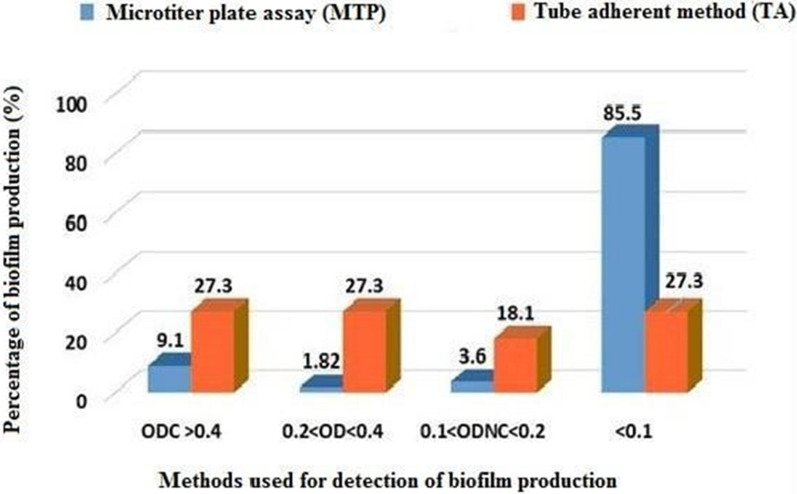
Results of biofilm production by tube adherent method (TA) and microtiter plate assay (MTP)

Regarding the qualitative analysis by the tube adherent method, *P. aeruginosa* isolates were categorized by two independent observers according to their capacity for biofilm formation as follow:15 (27.3%) were strongly adherent isolates; 15 (27.3%) were moderately adherent isolates; 10 (18.1%) were weakly adherent isolates; and 15 (27.3%) were non-adherent isolates. On the other hand, the quantitative analysis of biofilm production by the microtiter plate method using the ELISA plate reader revealed that: 5 (9.1%) were strongly adherent isolates, 1 (1.82%) were moderately adherent isolates, 2 (3.6%) were weakly adherent isolates, and 47 (85.5%) were non-adherent isolates (Table 5; Fig. 9). The results of Kappa statistics (Kappa 0.324) indicated that there is poor agreement between both methods used for the detection of biofilm production (Table 6).

**Table 5:** Results of biofilm formation abilities by MTP method using the ELISA plate reader at 560 nm

Cut-off value calculation of MTP assay	OD results for 55 samples using the ELISA plate reader at 560 nm (n = 55)	Biofilm formation ability
ODC > 0.4 (4 × ODc < ODi)	1 = 1.326 2 = 1.043 3 = 0.814 4 = 0.750 5 = 0.440	5 (9.1%) strongly adherent isolates
0.2 < OD < 0.4 (2 × ODc < ODi ≤ 4 × ODc),	0.374	1 (1.82%) moderately adherent isolates
0.1 < OD_NC_ < 0.2 (ODc < OD_i_ ≤ 2 × ODc),	1 = 0.134 2 = 0.114	2 (3.6%) weakly adherent isolates
< 0.1 (ODi < ODc)	0.064 and 0.001	47 (85.5%) nonadherent isolates

*MPA* Microtiter Plate Assay, *ODc* optical density of control, *ODi* optical density of the samples, *OD*_*NC*_ optical density of negative control

**Table 6:** Agreement between TA and MTP for biofilm formation based on Kappa analysis

Cut-of value calculation of MTP	Biofilm formation ability	Quantification of biofilm production by MTP		Tube method (TA)		p value	Kappa agreement
		N = 55	%	N = 55	%		
ODC > 0.4	Strong	5	9.1	15	27.3	< 0.001*	0.324
0.2 < OD < 0.4	Moderate	1	1.82	15	27.3		
0.1 < ODNC < 0.2	Weak	2	3.6	10	18.1		
< 0.1	No biofilm	47	85.5	15	27.3		

## Discussion

*P. aeruginosa* is an opportunistic bacterium that can cause localized or systemic illness in newly hatched chicks and growing poultry. Infections with *Pseudomonas* in birds are critical because outbreaks may spread quickly among poultry flocks, causing mortality at any age [28]. Additionally, zoonotic infections in humans may occur through the consumption of contaminated poultry meat and its by-products [53]. In the present study, 80 *P. aeruginosa* isolates were identified based on traditional methods of bacterial identification. The suspected isolates were then screened by PCR for identification of 16S rRNA. PCR successfully amplified the target amplicon in 55 (18.3%) isolates which were confirmed to be *P. aeruginosa.* The false-positive culture findings may be due to the overgrowth of other bacteria or the existence of non-cultivable or mutant microorganisms, making it difficult to confirm *P. aeruginosa* by traditional methods. Similarly, Qin et al. [54] reported that the identification of *P. aeruginosa* with traditional methods takes a long time, expensive, and needs more technicians. As a result, advanced molecular techniques for the diagnosis of the infection have been developed. The small subunit ribosomal RNA (16S rRNA), which is highly conserved and rarely changes among species, is rapidly becoming a primary tool for phylogenetic analysis and species classification [55]. However, Pseudomonas typing by informative typing methods such as multilocus sequence typing (MLST) is very essential which can also be used to investigate the population structure of bacterial populations at different levels to understand the transmission route of this bacteria.

Hatcheries are prone to be a reservoir for infectious agents due to their critical role in the poultry production chain [56]. The problem often begins with contaminated eggs that are incubated in conditions optimal for microbial growth. Infection of the yolk sac and death-in-shell occur in chicks just before hatching, resulting in decreased hatchability and higher mortality [57]. In this study, *P. aeruginosa* was confirmed in 20% of dead in-shell chicks. This result is in agreement with the results of many previous studies from Egypt [58, 59] that reported a similar frequency (20% and 19%, respectively) of *P. aeruginosa* in dead in-shell chicks. While, a higher isolation rate (52%) was recorded by Elsayed et al. [60]. On the other hand, a lower prevalence (1.2%) was recorded by Amer et al. [61]. The infection may enter the flock of birds from a variety of sources due to mistakes in management and industrial procedures or mechanically through wounded skin or contaminated needles during the vaccination process [62]. The microclimate of the chicken house is a suitable medium for the development of *P. aeruginosa* infection, which can persist for a long period in chicken farms due to the emergence of resistance to several antibiotic treatments and traditional disinfectants [63].

Regarding diseased and freshly dead broiler chick samples, the isolation rate of *P. aeruginosa* was 17.5% (35/200). Similarly, Mohamed [64] and Bakheet et al. [65] recorded 17.6% and 18.6% isolation rates respectively from one-day old chicks.**.** The higher prevalence of *P. aeruginosa* indicates higher environmental pollution and a reduction in biosecurity programmes used on poultry farms. As a result, it is essential to avoid *Pseudomonas* invasions on poultry farms. Farm management should take extreme caution in the presence of any possible source of contamination. Workers should be trained on how to prevent infectious diseases brought on by the environment, as well as disinfectants should be used on a regular basis in hatcheries, incubators, and house environments.

*P. aeruginosa* possesses a large variety of extracellular and cellular virulence factors that are involved in its pathogenicity [63]. In this study, *las*B, *tox*A, and *exo*S, were detected in all *P. aeruginosa* isolates. Similar results were reported in a previously published study by Wei et al. [66], who detected the *las*B gene in 100% of *P. aeruginosa* isolates from drinking water in China, while the percentages of *exo*S and *tox*A genes were 86.3% and 89.4%, respectively. In Egypt, studies by Hassan et al. [67] and Tartor et al. [68] detected the *tox*A gene in 100% of *P. aeruginosa* isolates obtained from broiler chickens. Similarly, Radwan et al. [69] and Shahat et al. [59] have reported higher frequencies of *P. aeruginosa* virulence-associated genes from broiler chickens in Egypt. The predominance of *tox*A, *las*B, and *exo*S in *P. aeruginosa* clinical isolates is associated with a significant role for these virulence factors in chicken respiratory illnesses, which could be highlighted as potential therapeutic targets for treating infections caused by *P. aeruginosa*-resistant strains [68].

Antibiotic resistance in *P. aeruginosa* is increasing globally. In Egypt and other developing countries, medical waste that includes active pharmaceutical compounds from antibiotic manufacturing facilities is released into rivers or the environment [70]. Furthermore, there is a scarcity of information on the extent to which antimicrobials are used in poultry farms, whether as growth promoters or as therapeutic or prophylactic medicines. This may be the hotspot for being infected with resistant organisms, which are expected to pose a significant threat to public health [70]. Unfortunately, there are no laws in Egypt that govern the use of antibiotics in poultry farms, so the improper use of antimicrobials results in a rapid selection of multi resistant strains in poultry and plays an important role in the spread of antibiotic-resistant bacteria from animals to humans [71] In this study, the results of antimicrobial susceptibility testing proved that the isolates obtained from broiler chicks and dead in-shell chicks displayed resistance to the most common antibiotic family used mainly in Egyptian poultry farms either as therapeutic drugs or growth promotion agents to speed up the growth based on the review performed by Kimera et al. [72]. In this study, the antimicrobial sensitivity test of *P. aeruginosa* isolates showed complete resistance to penicillin, amoxicillin, ceftriaxone, ceftazidime, streptomycin, erythromycin, spectinomycin, and doxycycline, followed by amoxicillin-clavulanic, kanamycin, and cefuroxime. These findings are consistent with the results from other studies in Egypt, which reported that the *P. aeruginosa* isolates from chicken showed 100% resistance to doxycycline, amoxicillin, and erythromycin [59, 73]. Additionally, Tawakol et al. [74] found that *P. aeruginosa* isolates were resistant to doxycycline, penicillin, ceftazidime, and streptomycin. Furthermore, a lower resistance (45.4%, 41.82%, and 29.1%) to ceftazidime, cefotaxime, and ceftriaxone was reported by Abd El–Dayem et al. [57].

In the current study, a high sensitivity was observed to meropenem, imipenem, colistin sulfate, ciprofloxacin, and gentamicin. The high sensitivity of the studied isolates to imipenem and meropenem agreed with Hassan et al. [67] who recorded a higher sensitivity of *P. aeruginosa* isolates to imipenem and meropenem. This higher sensitivity of *P. aeruginosa* isolates to carbapenems may be explained by the minimal use of these antibiotics in routine treatments of poultry disease in Egypt. While, other studies [75, 76] reported a higher resistance of *P. aeruginosa* to carbapenems (imipenem and meropenem). The variations among the results may be attributable to defects in many conditions surrounding hatcheries or the extensive overuse of antibiotics for preventing and controlling bacterial infection in Egyptian poultry farms, or they may be a result of hypermutation, which occurs frequently in *P. aeruginosa* strains and leads to the development of various antimicrobial resistance [77]. Therefore, meropenem, imipenem, colistin sulfate, and ciprofloxacin were shown to be the most sensitive antibiotics, which appeared to be a potential therapy for *Pseudomonas* infection [78].

Noticeably, all the tested *P. aeruginosa* isolates were MDR, as they resisted one or more agents of at least six different classes, and the MDRI ranged from 0.5 to 0.8. This study finding is higher than the findings of previous studies [79, 80] which found lower frequencies of MDR *P. aeruginosa* isolates (75.8% and 56%, respectively).

Colistin is considered a last-resort antibiotic that is often used to treat multidrug-resistant *P. aeruginosa* strains [81]. Interestingly, in this study 10 strains (18.2%) showed resistance to colistin by the broth microdilution method (BMD). According to recent studies, *P. aeruginosa* is growing more resistant to colistin, which is considered one of the most severe global threats to human and veterinary medicine [22].

Studying the antimicrobial resistance of *P. aeruginosa* isolates obtained from broiler chicks and dead in-shell chicks in regards to their significance to public health revealed that all isolates showed complete resistance towards penicillin, amoxicillin, ceftriaxone, ceftazidime, streptomycin, and erythromycin with percentages of 100% for each, followed by amoxicillin-clavulanic, kanamycin, cefuroxime, gentamicin, amikacin, apramycin, and colistin sulfate, which are all classified by the World Health Organization [52] as a critical antibiotic for human consumption (Category I). Furthermore, complete resistance was observed for highly important antibiotics: doxycycline (100%) (Category II), as well as complete resistance to spectinomycin was found in all isolates (100%), which was identified as an important drug for human medicine (Category III). In many countries, the majority of the antibiotics used for poultry production and treatment are also used as critical medications for humans. The misuse of antibiotics in food animal production is among the most significant contributors to the global rise and spread of antibiotic resistance. As a result, it is recommended to reduce the use of essential antibiotics, especially those that are critical. Furthermore, proper antibiotic use and the development of scientific monitoring systems are the most effective ways to reduce the adverse consequences of antibiotic misuse and ensure the safety of animal-derived food [82]. So, more research is needed to create more efficient and safe antibiotic alternatives to sustain a healthy agricultural economy and preserve potent antibiotics for successful therapy in humans.

In this study, ESBL production was confirmed in 12 (21.8%) and 15 isolates (27.3%) by DDST and PCDDT respectively, but the two tests were unable to confirm ESBL production in the remaining isolates, it might be due to non-enzymatic mechanisms, as this non-enzymic resistance isolates lakes the transferable β-lactamase or generates low quantities of cephalosporinase (100 mU/mg protein without induction) but contains numerous resistance mechanisms such as decreased permeability, hyperactivity of several efflux systems, poor cephalosporinase synthesis, and altered penicillin-binding protein sensitivity. The majority of non-enzymic resistant isolates were categorized as having intermediate resistance to β-lactams [83].

In this study, DDST was less sensitive than PCDDT. This result is similar to that reported by Paterson et al. [84], who reported that PCDDT is more effective in detecting the ESBLs than DDST, it supposed that DDST lacks sensitivity because of the problem of optimal disc space. This study result is similar to the result obtained by Gupta et al. [85], who reported that 26.47% of *P. aeruginosa* isolates were found to be ESBL producers by DDST and 29.4% of isolates by PCDDT. Noticeably, the most common genotype for ESBL production was *bla*_TEM_ (72.7%), followed by *bla*_SHV_ (69.1%), and *bla*_CTX-M_ (54%). The majority of the *P. aeruginosa* isolates were positive for the *bla*_TEM_ gene, which may be due to the incorrect use of antimicrobial medications such as broad-spectrum β-lactams, which results in mutations of β-lactam genes, particularly *bla*_TEM_. The current study findings were consistent with those of Ohore et al. [86], who found *P. aeruginosa* isolates from Nigeria were positive for TEM (64%), SHV (52%), and CTX-M(44%). In South Africa Hosu et al. [87] detected *bla*_TEM_ in 79.3% of *P. aeruginosa* strains, followed by *bla*_SHV_ (69.5%) and *bla*_CTX-M_ (31.7%). In Egypt, Ahmed et al. [88] detected the *bla*_TEM_ gene in 80% of *P. aeruginosa* isolates obtained from broiler chicks.

Regarding the *bla*_OXA-10_ gene, it was found in 9 isolates (16.4%). This result is similar to that obtained by Lee et al. [89], who detected *bla*_OXA-10_ in 13.1% of tested *P. aeruginosa* isolates in Korea, while, higher results were obtained by Alipour et al. [38], with percentages of 63%. In contrast, Amirkamali et al. [90] reported that all isolates were negative for *bla*_OXA-10_. Interestingly, this is the first report for the detection of *bla*_OXA-10_ in *P. aeruginosa* from Egypt. Obviously, all isolates were negative for both the *bla*_OXA-2_ gene and *bla*_VEB-1_ gene.

Broad-spectrum enzymes such as TEM, SHV, CTX-M, and OXA-type β-lactamases such as OXA-10 and OXA-2 are responsible for the resistance to penicillins, first-, second-, and third-generation cephalosporins such as cefotaxime, cefuroxime, ceftriaxone, and ceftazidime, and they have recently developed resistance to clavulanic acid but not to carbapenems and cephamycins [91]. In this study, the genotypic detection of ESBL genes revealed that the *bla*_TEM,_ *bla*_SHV_*, bla*_CTX-M_*, and bla*_OXA-10_ genes were detected in 72.7%, 69.1%, 54%, and 16.4% of the isolates, respectively. Furthermore, the phenotypic antimicrobial susceptibility testing revealed that all isolates were completely resistant to penicillin, amoxicillin, ceftriaxone, ceftazidime. In addition, high resistance to amoxicillin-clavulanic acid and cefuroxime was observed, as well as intermediate resistance to cefotaxime. The results of genotypic and phenotypic detection of ESBL isolates confirm the relationship between the presence of ESBL genes (*bla*_TEM_*, bla*_SHV_*, bla*_CTX-M_*, and bla*_OXA-10_) and resistance to β-lactam antibiotics and cephalosporins.

Integron has a significant role in the spread of MDR Gram-negative bacteria, particularly *Pseudomonas*. Generally, *Int*1 has been identified as the main source of resistance genes, as it harbors genes those encode extended-spectrum β-lactamases and metallo β-lactamases, which hydrolyze third- and fourth-generation cephalosporins and carbapenems [19]. The genetic detection of the *int*1 gene in this study revealed that the *int*1 gene was identified in 87.3% (48/55) of the tested isolates. This result is consistent with the findings obtained by Maclean et al. [92] who reported a detection rate of 77.3%. The lowest frequencies were reported in Brazil (41.5%), Spain (46.4%), and southern China (45.8%) by Fonseca et al. [93], Ruiz-Martínez et al. [94], and Xu et al. [95], respectively. The higher detection rate of integron might be attributable to a recent trend of rapidly growing integron-positive rates among *P. aeruginosa* clinical isolates in Egypt.

Markedly, there is a high correlation between the presence of *int*-1 gene and the resistance to β-lactam antibiotics, and streptomycin [96]. Interestingly, there is a high antimicrobial resistance among isolates that were positive for the *Int*1 gene and also showed complete resistance to β-lactam antibiotics and streptomycin, which seems to play an important role in MDR among these isolates. On the other hand, there were seven MDR isolates lack integrons. It may be due to other mechanisms, such as low outer membrane permeability, the expression of efflux pumps, and the production of antibiotic-inactivating enzymes, that make these isolates resistant to antibiotics. However, sequencing of Integrons integrate mobile gene cassettes is essential for surveillance of antibiotic resistance determinants.

The *mcr*-1 gene was found in 18.2% (10/55) of the *P. aeruginosa* isolates tested in this study. These isolates showed resistance to colistin in the broth microdilution test. To our knowledge, this study is the first report on the genetic detection of the *mcr*-1 gene in *P. aeruginosa* clinical isolates from dead in shell chicks and broiler chicks in Egypt. However, the other *mcr* gene variants is essential to be investigated. This high percentage of *mcr-*1 resistant genes is cause for concern, as Egypt has a high prevalence of infectious diseases and no restrictions on antibiotic use in veterinary and human medicine. It also predicts the emergence of difficult-to-cure diseases in Egypt due to the possible transmission of colistin resistance to highly resistant bacteria [97]. As a result, proper colistin usage and the establishment of scientific monitoring systems are the best strategies to limit colistin resistance. More research is needed to develop more efficient and safe antibiotic alternatives to support a healthy agricultural economy and conserve potent antibiotics for successful human therapy.

In this study, the ability of *P. aeurginosa* to produce biofilms was evaluated by using two different assays, the tube adherent method, and the microtiter plate method. Based on the results of this study, MTP is regarded as a more reliable and accurate assay than the TA method. The agreement between biofilm formation for both independent methods (TA and MTP) measured through Kappa statistics revealed poor agreement between the two tests and that it is also statistically significant which were agreed with previous studies [98, 99].

Biofilm in poultry farms aids in the acquisition of new genes for virulence and environmental survival, so it is necessary to develop a control strategy to reduce the impact of biofilm formation by *P. aeruginosa* [100]. Producers and veterinarians can take preventive and control measures like cleaning the entire drinking system after antibiotic treatment, developing biofilm inhibitors based on knowledge of the molecular process of biofilm formation, and using disinfectants like hydrogen peroxide and sodium hypochlorite-based products, which have strong bactericidal effects against *P. aeruginosa* biofilms [101].

## Conclusions

In conclusion, the high prevalence of virulent *P.aeruginosa* emphasizes the importance of paying closer attention to the risk of bacterial contamination, the potential effect on hatchability, and the health of one-day-old chicks. To prevent economic losses, sanitary precautions should be created in the chicken manufacturing organization. *P. aeruginosa* isolates have shown significant resistance to a variety of antibiotics commonly used in Egyptian chicken farms such as penicillin, amoxicillin, streptomycin, erythromycin, spectinomycin, and amoxicillin-clavulanic. Therefore, a strong antibiotic strategy and the implementation of infection control initiatives will aid in the reduction of *P. aeruginosa* resistance. Although the results of this study provide a comprehensive approach, combining cultural, molecular, and phenotypic methods, adds value to our understanding of the epidemiology and potential risks associated with *P. aeruginosa* infections in poultry*,* while a further studies are very recommended for detection of the other *mcr* variants, sequencing of Integrons integrate mobile gene cassettes, and *Pseudomonas* typing using Multlocus sequence typing (MLST).

### Supplementary Information

**Additional file 1: Figure S1**. Agarose gel electrophoresis showing amplification a 956bp fragment of 16S rRNA gene of *P. aeruginosa* isolates `. L: 100 bp ladder. 9: Control negative. 10: Control positive. **Table S1.** The interpretation of *P. aeruginosa* sensitivity test according to (CLSI/NCCLS, 2019). **Table S2**: Antimicrobial Resistance Patterns and Antibiotypes of *P. aeruginosa* isolates.

## Data Availability

The data that support the findings of this study are available from the corresponding author upon reasonable request.

## References

[1] Abd El-Ghany WA (2021). *Pseudomonas aeruginosa* infection of avian origin: zoonosis and one health implications. Vet World.

[2] Dégi J, Moțco O-A, Dégi DM, Suici T, Mareș M, Imre K (2021). Antibiotic susceptibility profile of *Pseudomonas aeruginosa* canine isolates from a multicentric study in Romania. Antibiotics.

[3] Hai-ping HE (2009). Isolation and identify of *Pseudomonas aeruginosa* in chicken dead-embryos. Chin Qinghai J Anim Vet Sci.

[4] Dinev I, Denev S, Beev G (2013). Clinical and morphological studies on spontaneous cases of *Pseudomonas aeruginosa* infections in birds. Pak Vet J.

[5] Wagner VE, Filiatrault MJ, Picardo KF, Iglewski BH (2008). Pseudomonas aeruginosa virulence and pathogenesis issues. Pseud Genom Mol Biol.

[6] Urgancı NN, Yılmaz N, Koçer Alaşalvar G, Yıldırım Z (2022). *Pseudomonas aeruginosa* and its pathogenicity. Turkish J Agric Food Sci Technol.

[7] Michalska M, Wolf P (2015). Pseudomonas Exotoxin A: optimized by evolution for effective killing. Front Microbiol..

[8] Klein EY, Van Boeckel TP, Martinez EM, Pant S, Gandra S, Levin SA (2018). Global increase and geographic convergence in antibiotic consumption between 2000 and 2015. Proc Natl Acad Sci.

[9] Maged O, Hamdey E. The analysis of livestock industry frame in Egypt: proposal in the light of bird flu crisis. IDSC: Ministerial Cabinet Information and Designing Making Supporting Center: report. 2006;29(5):2006.

[10] Hedman HD, Vasco KA, Zhang L (2020). A review of antimicrobial resistance in poultry farming within low-resource settings. Animals.

[11] Bayoumi A, Zidan S, Sakr MA, ElMashtouly A, Hadad G (2023). Prevalence of extended spectrum Β-lactamase (ESBL) producing *Escherichia coli* and molecular characterization of ESBL, Carbapenemases, and Blacmy2 Genes in Broilers and Humans at Menoufia Governorate. Egypt J Curr Vet Res.

[12] Mohamed ES, Khairy RMM, Abdelrahim SS (2020). Prevalence and molecular characteristics of ESBL and AmpC β-lactamase producing Enterobacteriaceae strains isolated from UTIs in Egypt. Antimicrob Resist Infect Control.

[13] Ahmed AS, Nasef SA, El Enbaawy MI. Emergency of extended-spectrum beta-lactamase-producing pseudomonas aeruginosa isolated from broiler chickens in Egypt.

[14] Elmonir W, Abd El-Aziz NK, Tartor YH, Moustafa SM, Abo Remela EM, Eissa R (2021). Emergence of colistin and carbapenem resistance in extended-spectrum β-lactamase producing *Klebsiella pneumoniae* isolated from chickens and humans in Egypt. Biology.

[15] Boucher Helen W, Talbot George H, Bradley John S, Edwards John E, Gilbert D, Rice Louis B (2009). Bad bugs, no drugs: no ESKAPE! an update from the infectious diseases Society of America. Clin Infect Dis.

[16] Bush K, Jacoby GA, Medeiros AA (1995). A functional classification scheme for beta-lactamases and its correlation with molecular structure. Antimicrob Agents Chemother.

[17] Poole K (2005). Aminoglycoside resistance in *Pseudomonas aeruginosa*. Antimicrob Agents Chemother.

[18] Fluit AC, Schmitz FJ (2004). Resistance integrons and super-integrons. Clin Microbiol Infect.

[19] Weldhagen GF (2004). Integrons and β-lactamases—a novel perspective on resistance. Int J Antimicrob Agents.

[20] Ahmed ZS, Elshafiee EA, Khalefa HS, Kadry M, Hamza DA (2019). Evidence of colistin resistance genes (mcr-1 and mcr-2) in wild birds and its public health implication in Egypt. Antimicrob Resist Infect Control..

[21] Al-Kadmy IMS, Ibrahim SA, Al-Saryi N, Aziz SN, Besinis A, Hetta HF (2020). Prevalence of Genes Involved in Colistin Resistance in *Acinetobacter baumannii:* first report from Iraq. Microb Drug Resist.

[22] Tehrani S, Samami H, Keyvanfar A, Hashemi A (2022). Detection of carbapenems and colistin resistance genes in *Pseudomonas aeruginosa* and *Acinetobacter baumannii*: a single-center study in Iran. Nov Biomed.

[23] Yuan Y, Qu K, Tan D, Li X, Wang L, Cong C (2019). Isolation and characterization of a bacteriophage and its potential to disrupt multi-drug resistant *Pseudomonas aeruginosa* biofilms. Microb Pathog.

[24] Parsek MR, Singh PK (2003). Bacterial biofilms: an emerging link to disease pathogenesis. Annu Rev Microbiol.

[25] Vestby LK, Grønseth T, Simm R, Nesse LL (2020). Bacterial biofilm and its role in the pathogenesis of disease. Antibiotics.

[26] Balcázar JL, Subirats J, Borrego CM (2015). The role of biofilms as environmental reservoirs of antibiotic resistance. Front Microbiol.

[27] Jaksch W. Euthanasia of day-old male chicks in the poultry industry. 1981.

[28] Shukla and Mishra (2015). *Pseudomonas aeruginosa* infection in broiler chicks in Jabalpur. Int J Ext Res.

[29] Quinn PJ, Markey BK, Leonard FC, Hartigan P, Fanning S, Fitzpatrick E (2011). Veterinary microbiology and microbial disease.

[30] Ramadan H, Awad A, Ateya A (2016). Detection of phenotypes, virulence genes and phylotypes of avian pathogenic and human diarrheagenic *Escherichia coli* in Egypt. J Infect Dev Countries.

[31] Spilker T, Coenye T, Vandamme P, LiPuma JJ (2004). PCR-based assay for differentiation of *Pseudomonas aeruginosa* from other Pseudomonas species recovered from cystic fibrosis patients. J Clin Microbiol.

[32] Tamura K, Stecher G, Peterson D, Filipski A, Kumar S (2013). MEGA6: molecular evolutionary genetics analysis version 6.0. Mol Biol Evol..

[33] Younis G (2015). Extracellular enzymes and toxins of *Pseudomonas aeruginosa* strains isolated from clinically diseased Egyptian cows. Adv Animal Vet Sci.

[34] Lévesque C, Piché L, Larose C, Roy PH (1995). PCR mapping of integrons reveals several novel combinations of resistance genes. Antimicrob Agents Chemother.

[35] Colom K, PÃ©rez J, Alonso R, FernÃ¡ndez-Aranguiz A, LariÃo E, Cisterna RN. Simple and reliable multiplex PCR assay for detection of blaTEM, blaSHV and blaOXA-1 genes in Enterobacteriaceae. FEMS Microbiol Lett. 2003;223(2):147–51. 10.1016/S0378-1097(03)00306-9 12829279

[36] Archambault M, Petrov P, Hendriksen RS, Asseva G, Bangtrakulnonth A, Hasman H (2006). Molecular characterization and occurrence of extended-spectrum β-lactamase resistance genes among *Salmonella enterica* serovar corvallis from Thailand, Bulgaria, and Denmark. Microb Drug Resist.

[37] Mirsalehian A, Feizabadi M, Nakhjavani FA, Jabalameli F, Goli H, Kalantari N (2010). Detection of VEB-1, OXA-10 and PER-1 genotypes in extended-spectrum β-lactamase-producing *Pseudomonas aeruginosa* strains isolated from burn patients. Burns.

[38] Alipour T, Sadeghifard N, Amirmozafari N, Ghafurian S, Abdulamir AS, Mohebi R (2010). Incidence of extended spectrum beta-lactamase producing *Pseudomonas aeruginosa* and frequency of oxa-2 and oxa-10 genes. Aust J Basic Appl Sci.

[39] Liu Y-Y, Wang Y, Walsh TR, Yi L-X, Zhang R, Spencer J (2016). Emergence of plasmid-mediated colistin resistance mechanism MCR-1 in animals and human beings in China: a microbiological and molecular biological study. Lancet Infect Dis.

[40] Koneman EW, Allen SD, Janda WM, Schreckenberger PC, Winn WC (1997). Diagnostic microbiology. The nonfermentative gram-negative bacilli.

[41] Bauer AW, Kirby WMM, Sherris JC, Turck M (1966). Antibiotic susceptibility testing by a standardized single disk method. Am J Clin Pathol..

[42] CLSI. What’s new in the 2019 CLSI standards for antimicrobial susceptibility testing (AST). 2019.

[43] Magiorakos AP, Srinivasan A, Carey RB, Carmeli Y, Falagas ME, Giske CG (2012). Multidrug-resistant, extensively drug-resistant and pandrug-resistant bacteria: an international expert proposal for interim standard definitions for acquired resistance. Clin Microbiol Infect.

[44] Chandran A, Hatha AAM, Varghese S, Sheeja KM (2008). Prevalence of multiple drug resistant *Escherichia coli* serotypes in a tropical estuary, India. Microb Environ.

[45] Eucast T. European Committee on Antimicrobial Susceptibility Testing, Breakpoint tables for interpretation of MICs and zone diameters. European Society of Clinical Microbiology and Infectious Diseases Basel; 2015.

[46] Lin Z, Zhao X, Huang J, Liu W, Zheng Y, Yang X (2019). Rapid screening of colistin-resistant *Escherichia coli*, *Acinetobacter baumannii* and *Pseudomonas aeruginosa* by the use of Raman spectroscopy and hierarchical cluster analysis. Analyst.

[47] Jarlier V, Nicolas MH, Fournier G, Philippon A (1988). Extended broad-spectrum-lactamases conferring transferable resistance to newer-lactam agents in enterobacteriaceae: hospital prevalence and susceptibility patterns. Clin Infect Dis.

[48] Ahmed OI, El-Hady SA, Ahmed TM, Ahmed IZ (2013). Detection of bla SHV and bla CTX-M genes in ESBL producing *Klebsiella pneumoniae* isolated from Egyptian patients with suspected nosocomial infections. Egypt J Med Human Genet.

[49] Singhai M, Malik A, Shahid M, Malik MA, Goyal R (2012). A study on device-related infections with special reference to biofilm production and antibiotic resistance. J Glob Infect Dis.

[50] StepanoviĆ S, VukoviĆ D, Hola V, Bonaventura GD, DjukiĆ S, ĆIrkoviĆ I, et al. Quantification of biofilm in microtiter plates: overview of testing conditions and practical recommendations for assessment of biofilm production by staphylococci. APMIS. 2007;115(8):891–9. 10.1111/j.1600-0463.2007.apm_630.x 17696944

[51] Vasudevan P, Nair MKM, Annamalai T, Venkitanarayanan KS (2003). Phenotypic and genotypic characterization of bovine mastitis isolates of *Staphylococcus aureus* for biofilm formation. Vet Microbiol.

[52] World Health Organization. Critically important antimicrobials for human medicine: ranking of antimicrobial agents for risk management of antimicrobial resistance due to non-human use. 2017.

[53] Morales PA, Aguirre JS, Troncoso MR, Figueroa GO (2016). Phenotypic and genotypic characterization of Pseudomonas spp. present in spoiled poultry fillets sold in retail settings. LWT..

[54] Qin X, Emerson J, Stapp J, Stapp L, Abe P, Burns JL (2003). Use of real-time PCR with multiple targets to identify *Pseudomonas aeruginosa* and other nonfermenting gram-negative bacilli from patients with cystic fibrosis. J Clin Microbiol.

[55] Woese CR (1987). Bacterial evolution. Microbiol Rev.

[56] Wales A, Davies R (2020). Review of hatchery transmission of bacteria with focus on Salmonella, chick pathogens and antimicrobial resistance. Worlds Poult Sci J.

[57] Abd El-Dayem GA, Ramadan AH, Ali HS. Egypt J Anim Health. 2021:80–99.

[58] Azmy RW. Some studies on bacterial agants causing embryonic mortalities in chickens and ducks. 2010.

[59] Shahat H, Mohamed H, Abd Al-Azeem M, Nasef S (2019). Molecular detection of some virulence genes in *Pseudomonas aeruginosa* isolated from chicken embryos and broilers with regard to disinfectant resistance. SVU-Int J Vet Sci.

[60] Elsayed MSA, Ammar AM, Al Shehri ZS, Abd-ElRahman H (2016). Virulence repertoire of pseudomonas aeruginosa from some poultry farms with detection of resistance to various antimicrobials and plant extracts. Cell Mol Biol..

[61] Amer MM, Elbayoumi KM, Amin Girh ZM, Mekky HM, Rabie NS (2017). A study on bacterial contamination of dead in shell chicken embryos and culled one day chicks. Int J Pharm Phytopharmacol Res.

[62] John Barnes H. Other bacterial disease: pseudomonas. In: Calnek, BW, John Barnes, H, Beard, CW, Mcdougald LR, Saif YM, editors. Diseases of poultry. 1997;10:291–2.

[63] Kebede F (2010). Pseudomonas infection in chickens. J Vet Med Anim Health.

[64] Mohamed HA (2004). Some studies on Pseudomonas species in chicken embryos and broilers in Assiut governorate. Ass Univ Bull Environ Res.

[65] Bakheet AA, Naglaa MA, Sayed AH, Soad AN (2017). Detection of disinfectant resistant aerobic bacteria in unhatched chicken eggs. Benha Vet Med J.

[66] Wei L, Wu Q, Zhang J, Guo W, Gu Q, Wu H (2020). Prevalence, virulence, antimicrobial resistance, and molecular characterization of *Pseudomonas aeruginosa* isolates from drinking water in China. Front Microbiol..

[67] Hassan WH, Ibrahim AMK, Shany SAS, Salam HSH (2020). Virulence and resistance determinants in *Pseudomonas aeruginosa* isolated from pericarditis in diseased broiler chickens in Egypt. J Adv Vet Anim Res.

[68] Tartor YH, El-Naenaeey EY (2016). RT-PCR detection of exotoxin genes expression in multidrug resistant *Pseudomonas aeruginosa*. Cell Mol Biol (Noisy-le-grand).

[69] Radwan IAE, Shehata AH, Abed A, Reda Hosni A (2016). Bacterial species associated with broiler proventriculitis and antimicrobial resistance of clinical important species. J Vet Med Res..

[70] Larsson DGJ (2014). Pollution from drug manufacturing: review and perspectives. Philos Trans R Soc Lond B Biol Sci.

[71] Moawad AA, Hotzel H, Neubauer H, Ehricht R, Monecke S, Tomaso H (2018). Antimicrobial resistance in Enterobacteriaceae from healthy broilers in Egypt: emergence of colistin-resistant and extended-spectrum β-lactamase-producing *Escherichia coli*. Gut Pathogens.

[72] Kimera ZI, Mshana SE, Rweyemamu MM, Mboera LEG, Matee MIN (2020). Antimicrobial use and resistance in food-producing animals and the environment: an African perspective. Antimicrob Resist Infect Control.

[73] Eraky RD, Abd El-Ghany WA, Soliman KM (2020). Studies on *Pseudomonas aeruginosa* infection in hatcheries and chicken. J Hellenic Vet Med Soc.

[74] Tawakol M, Nabil N, Reda R (2018). Molecular studies on some virulence factors of *Pseudomonas aeruginosa* isolated from chickens as a biofilm forming bacteria. Assiut Vet Med J.

[75] Diab M, Fam N, El-Said M, El-Defrawy EE-DI, Saber M (2013). Occurrence of VIM-2 Metallo-ß-Lactamases in imipenem resistant and susceptible *Pseudomonas aeruginosa* clinical isolates from Egypt. Afr J Microbiol Res.

[76] Ejikeugwu C, Nworie O, Saki M, Al-Dahmoshi HOM, Al-Khafaji NSK, Ezeador C (2021). Metallo-β-lactamase and AmpC genes in *Escherichia coli*, *Klebsiella pneumoniae*, and *Pseudomonas aeruginosa* isolates from abattoir and poultry origin in Nigeria. BMC Microbiol..

[77] Maciá MD, Blanquer D, Togores B, Sauleda J, Pérez JL, Oliver A (2005). Hypermutation is a key factor in development of multiple-antimicrobial resistance in *Pseudomonas aeruginosa* strains causing chronic lung infections. Antimicrob Agents Chemother.

[78] Aniokette U, Iroha CS, Ajah MI, Nwakaeze AE (2016). Occurrence of multi-drug resistant Gram-negative bacteria from poultry and poultry products sold in Abakaliki. J Agric Sci Food Technol.

[79] Ahmed AS, Nasef SA, El Enbaawy MI. Emergency of extended-spectrum beta-lactamase-producing pseudomonas aeruginosa isolated from broiler chickens in Egypt. 2022.

[80] Hassuna NA, Mohamed AHI, Abo-Eleuoon SM, Rizk HA-WA. High prevalence of multidrug resistant *Pseudomonas aeru*. Arch Clin Microbiol. 2015;6(4).

[81] Dößelmann B, Willmann M, Steglich M, Bunk B, Nübel U, Peter S (2017). Rapid and consistent evolution of colistin resistance in extensively drug-resistant *Pseudomonas aeruginosa* during morbidostat culture. Antimicrob Agents Chemother.

[82] Mostafa Badr J, Reyad El Saidy F, Abdelwahed AA (2020). Emergence of multi-drug resistant Pseudomonas aeruginosa in broiler chicks. Int J Microbiol Biotechnol..

[83] Cavallo JD (2000). Antibiotic susceptibility and mechanisms of beta-lactam resistance in 1310 strains of *Pseudomonas aeruginosa*: a French multicentre study (1996). J Antimicrob Chemother.

[84] Paterson DL, Bonomo RA (2005). Extended-spectrum beta-lactamases: a clinical update. Clin Microbiol Rev.

[85] Gupta B, Sharma R, Garg K. Diagnostic characterisation of various phenotypic methods for class-a extended spectrum of β-lactamase among multidrug resistant pseudomonas aeruginosa isolated from diabetic patients. J Clin Diagn Res. 2022.

[86] Ohore HU, Akinduti PA, Ahuekwe EF, Ajayi AS, Olasehinde GI (2022). Molecular detection of ESBLs, TEM, SHV, and CTX-M in clinical *Pseudomonas aeruginosa* isolates in Ogun State. Bioenergy and biochemical processing technologies.

[87] Hosu MC, Vasaikar SD, Okuthe GE, Apalata T (2021). Detection of extended spectrum beta-lactamase genes in *Pseudomonas aeruginosa* isolated from patients in rural Eastern Cape Province, South Africa. Sci Rep..

[88] Ahmed AS, Nasef SA, El Enbaawy MI. Emergency of extended-spectrum beta-lactamase-producing *Pseudomonas aeruginosa* isolated from broiler chickens in Egypt. 2022:1–17.

[89] Lee S, Park Y-J, Kim M, Lee HK, Han K, Kang CS (2005). Prevalence of Ambler class A and D β-lactamases among clinical isolates of *Pseudomonas aeruginosa* in Korea. J Antimicrob Chemother.

[90] Amirkamali S, Naserpour-Farivar T, Azarhoosh K, Peymani A (2017). Distribution of the bla OXA, bla VEB-1, and bla GES-1 genes and resistance patterns of ESBL-producing *Pseudomonas aeruginosa* isolated from hospitals in Tehran and Qazvin. Iran Rev Soc Brasil Med Trop.

[91] Castanheira M, Simner PJ, Bradford PA (2021). Extended-spectrum β-lactamases: an update on their characteristics, epidemiology and detection. JAC-Antimicrob Resist..

[92] Maclean K, Njamo FOJP, Serepa-Dlamini MH, Kondiah K, Green E (2022). Antimicrobial susceptibility profiles among *Pseudomonas aeruginosa* isolated from professional SCUBA divers with otitis externa, swimming pools and the ocean at a diving operation in South Africa. Pathogens.

[93] Fonseca ÃRL, Vieira VNV, Cipriano RN, Vicente ACP (2005). Class 1 integrons in Pseudomonas aeruginosa isolates from clinical settings in Amazon region, Brazil. FEMS Immunol Med Microbiol..

[94] Ruiz-Martínez L, López-Jiménez L, Fusté E, Vinuesa T, Martínez JP, Viñas M (2011). Class 1 integrons in environmental and clinical isolates of *Pseudomonas aeruginosa*. Int J Antimicrob Agents.

[95] Xu Z, Li L, Shirtliff ME, Alam MJ, Yamasaki S, Shi L (2009). Occurrence and characteristics of class 1 and 2 integrons in *Pseudomonas aeruginosa* isolates from patients in southern China. J Clin Microbiol.

[96] Blahna MT, Zalewski CA, Reuer J, Kahlmeter G, Foxman B, Marrs CF (2006). The role of horizontal gene transfer in the spread of trimethoprim–sulfamethoxazole resistance among uropathogenic *Escherichia coli* in Europe and Canada. J Antimicrob Chemother.

[97] Khalifa HO, Ahmed AM, Oreiby AF, Eid AM, Shimamoto T, Shimamoto T (2016). Characterisation of the plasmid-mediated colistin resistance gene mcr-1 in *Escherichia coli* isolated from animals in Egypt. Int J Antimicrob Agents.

[98] Kunwar A, Shrestha P, Shrestha S, Thapa S, Shrestha S, Amatya NM (2021). Detection of biofilm formation among *Pseudomonas aeruginosa* isolated from burn patients. Burns Open.

[99] Nasirmoghadas P, Yadegari S, Moghim S, Esfahani BN, Fazeli H, Poursina F (2018). Evaluation of biofilm formation and frequency of multidrug-resistant and extended drug-resistant strain in *Pseudomonas aeruginosa* isolated from burn patients in Isfahan. Adv Biomed Res..

[100] Merino L, Procura F, Trejo FM, Bueno DJ, Golowczyc MA (2019). Biofilm formation by Salmonella sp. in the poultry industry: detection, control and eradication strategies. Food Res Int..

[101] Lineback CB, Nkemngong CA, Wu ST, Li X, Teska PJ, Oliver HF (2018). Hydrogen peroxide and sodium hypochlorite disinfectants are more effective against *Staphylococcus aureus* and *Pseudomonas aeruginosa* biofilms than quaternary ammonium compounds. Antimicrob Resist Infect Control..

