# Supplementary material for: Dissemination of mcr-1 and β-lactamase genes among Pseudomonas aeruginosa: molecular characterization of MDR strains in broiler chicks and dead-in-shell chicks infections
Source: Ann Clin Microbiol Antimicrob. 2024 Jan 28;23:9. doi: 10.1186/s12941-024-00669-4 (PMC10823725; doi:10.1186/s12941-024-00669-4)
Supplement: Supplementary file 1 — Additional file 1: Figure S1. Agarose gel electrophoresis showing amplification a 956bp fragment of 16S rRNA gene of P. aeruginosa isolates `. L: 100 bp ladder. 9: Control negative. 10: Control positive. Table S1. The interpretation of P. aeruginosa sensitivity test according to (CLSI/NCCLS, 2019). Table S2: Antimicrobial Resistance Patterns and Antibiotypes of P. aeruginosa isolates. [file 12941_2024_669_MOESM1_ESM.docx]

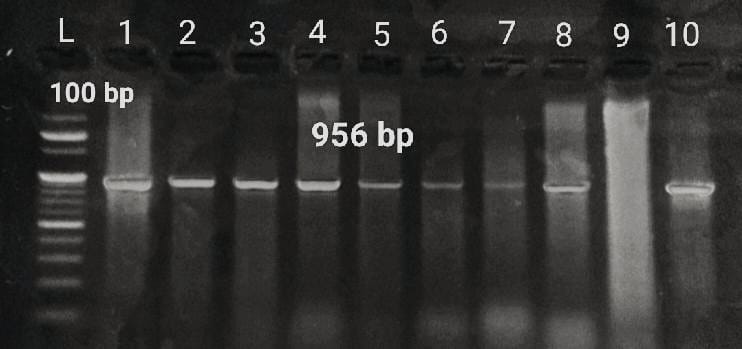


Figure S1. Agarose gel electrophoresis showing amplification a 956bp fragment of 16S rRNA gene of *P. aeruginosa* isolates. L: 100bp ladder. 9: Control negative. 10: Control positive.

**Table S1. The interpretation of *P. aeruginosa* sensitivity test according to (CLSI/NCCLS, 2019).**

| **Antimicrobial agent** | **Disc code** | **CPD** | **Zone diameter (mm)** | | |
| --- | --- | --- | --- | --- | --- |
|  |  |  | **Resistant**  **≥** | **Intermediate** | **Sensitive**  **≤** |
| **Penicillin** | **P** | **10U** | **28** | **__** | **29** |
| **Amoxicillin** | **AX** | **10μg** | **14** | **16-15** | **17** |
| **Amoxicillin-clavulanic** | **AMC** | **30μg** | **13** | **14-17** | **18** |
| **Imipenem** | **IPM** | **10μg** | **13** | **14-15** | **16** |
| **Meropenem** | **MEM** | **10μg** | **15** | **16-18** | **19** |
| **Ceftriaxone** | **CRO** | **30μg** | **13** | **14-20** | **21** |
| **Cefuroxime** | **CXM** | **30μg** | **14** | **15-17** | **18** |
| **Cefotaxime** | **CTX** | **30μg** | **14** | **15-22** | **23** |
| **Ceftazidime** | **CAZ** | **30μg** | **14** | **15-17** | **18** |
| **Gentamicin** | **CN** | **10μg** | **12** | **13-14** | **15** |
| **Streptomycin** | **S** | **10μg** | **11** | **12-13** | **14** |
| **Amikacin** | **AK** | **30μg** | **14** | **15-16** | **17** |
| **Kanamycin** | **K** | **30μg** | **13** | **14-17** | **18** |
| **Apramycin** | **APR** | **15μg** | **14** | **15-16** | **17** |
| **Colistinsulphate** | **CT** | **10μg** | **10** | **__** | **11** |
| **Erythromycin** | **E** | **15μg** | **13** | **14-22** | **23** |
| **Spectinomycin** | **SPT** | **100μg** | **14** | **15-17** | **18** |
| **Ciprofloxacin** | **CIP** | **5μg** | **15** | **16-20** | **21** |
| **Doxycycline** | **DO** | **30μg** | **12** | **13-15** | **16** |

Table S2: Antimicrobial Resistance Patterns and Antibiotypes of *P.aeruginosa* isolates:

| Antibiotypes | Resistance pattern | Isolates no (%) | No. of resistant  antibiotic | No. of resistant  antibiotic categories | MDRINDEX |
| --- | --- | --- | --- | --- | --- |
| I | P, AX, CRO, CAZ, S, E, SPT, DO, K | 1 | 9/19 | 6/10 | 0.5 |
| II | P, AX, CRO, CAZ, S, E, SPT, DO, CN | 1 | 9/19 | 6/10 | 0.5 |
| III | P, AX, CRO, CAZ, S, E, SPT, DO, AMC | 2 | 9/19 | 6/10 | 0.5 |
| IV | P, AX, CRO, CAZ, S, E, SPT, DO, AMC, CXM | 4 | 10/19 | 6/10 | 0.5 |
| V | P, AX, CRO, CAZ, S, E, SPT, DO, AMC, K | 2 | 10/19 | 6/10 | 0.5 |
| VI | P, AX, CRO, CAZ, S, E, SPT, DO, AMC, CT | 1 | 10/19 | 7/10 | 0.5 |
| VII | P, AX, CRO, CAZ, S, E, SPT, DO, CT, CXM | 1 | 10/19 | 7/10 | 0.5 |
| VIII | P, AX, CRO, CAZ, S, E, SPT, DO,K,APR | 1 | 10/19 | 6/10 | 0.5 |
| IX | P, AX, CRO, CAZ, S, E, SPT, DO, AMC,K, AK | 1 | 11/19 | 6/10 | 0.6 |
| X | P, AX, CRO, CAZ, S, E, SPT, DO, AMC, K, CXM | 5 | 11/19 | 6/10 | 0.6 |
| XI | P, AX, CRO, CAZ, S, E, SPT, DO, AMC, APR, CT | 1 | 11/19 | 7/10 | 0.6 |
| XII | P, AX, CRO, CAZ, S, E, SPT, DO, AMC, K, CN | 2 | 11/19 | 6/10 | 0.6 |
| XIII | P, AX, CRO, CAZ, S, E, SPT, DO, AMC, CN, CIP | 1 | 11/19 | 7/10 | 0.6 |
| XIV | P, AX, CRO, CAZ, S, E, SPT, DO, AMC, CXM,CT | 2 | 11/19 | 7/10 | 0.6 |
| XV | P, AX, CRO, CAZ, S, E, SPT, DO, AMC, CXM, AK | 1 | 11/19 | 6/10 | 0.6 |
| XVI | P, AX, CRO, CAZ, S, E, SPT, DO, AMC, CTX, CT | 1 | 11/19 | 7/10 | 0.6 |
| XVII | P, AX, CRO, CAZ, S, E, SPT, DO, K, APR, CT | 1 | 11/19 | 7/10 | 0.6 |
| XVIII | P, AX, CRO, CAZ, S, E, SPT, DO,CN, K, APR | 1 | 11/19 | 6/10 | 0.6 |
| XIX | P, AX, CRO, CAZ, S, E, SPT, DO, AK, K, APR | 1 | 11/19 | 6/10 | 0.6 |
| XX | P, AX, CRO, CAZ, S, E, SPT, DO, AMC, CN, AK, APR | 1 | 12/19 | 6/10 | 0.6 |
| XXI | P, AX, CRO, CAZ, S, E, SPT, DO, AMC, CXM, CN, K | 1 | 12/19 | 6/10 | 0.6 |
| XXII | P, AX, CRO, CAZ, S, E, SPT, DO, AMC, K, APR, CT | 1 | 12/19 | 7/10 | 0.6 |
| XXIII | P, AX, CRO, CAZ, S, E, SPT, DO, AMC, CXM, AK, APR | 1 | 12/19 | 6/10 | 0.6 |
| XXIV | P, AX, CRO, CAZ, S, E, SPT, DO, AMC, CXM, CTX, K | 1 | 12/19 | 6/10 | 0.6 |
| XXV | P, AX, CRO, CAZ, S, E, SPT, DO, CN, AK, K, APR | 2 | 12/19 | 6/10 | 0.6 |
| XXVI | P, AX, CRO, CAZ, S, E, SPT, DO,CXM, CN, K, APR | 1 | 12/19 | 6/10 | 0.6 |
| XXVII | P, AX, CRO, CAZ, S, E, SPT, DO, AMC, CXM, AK, K | 1 | 12/19 | 6/10 | 0.6 |
| XXVIII | P, AX, CRO, CAZ, S, E, SPT, DO, CXM, CTX, AK, K,APR | 1 | 13/19 | 6/10 | 0.7 |
| XXIX | P, AX, CRO, CAZ, S, E, SPT, DO, AMC, CXM, APR, CT, CIP | 2 | 13/19 | 8/10 | 0.7 |
| XXX | P, AX, CRO, CAZ, S, E, SPT, DO, AMC, CXM, K, CT, CIP | 1 | 13/19 | 8/10 | 0.7 |
| XXXI | P, AX, CRO, CAZ, S, E, SPT, DO, AMC, CN, AK, K, APR | 1 | 13/19 | 6/10 | 0.7 |
| XXXII | P, AX, CRO, CAZ, S, E, SPT, DO, AMC, CXM, CN, AK, K | 1 | 13/19 | 6/10 | 0.7 |
| XXXIII | P, AX, CRO, CAZ, S, E, SPT, DO, AMC, CXM,CN, APR, CT | 1 | 13/19 | 7/10 | 0.7 |
| XXXIV | P, AX, CRO, CAZ, S, E, SPT, DO, AMC, CXM, K, APR, CT | 1 | 13/19 | 7/10 | 0.7 |
| XXXV | P, AX, CRO, CAZ, S, E, SPT, DO, AMC, CXM, CTX, AK, K | 1 | 13/19 | 6/10 | 0.7 |
| XXXVI | P, AX, CRO, CAZ, S, E, SPT, DO, AMC, CXM, CN, K, CT, CIP | 1 | 14/19 | 8/10 | 0.7 |
| XXXVII | P, AX, CRO, CAZ, S, E, SPT, DO, AMC, CN, AK, K, APR, CT | 1 | 14/19 | 7/10 | 0.7 |
| XXXVIII | P, AX, CRO, CAZ, S, E, SPT, DO, AMC, CXM,CN, AK, K, APR | 1 | 14/19 | 6/10 | 0.7 |
| XXXIX | P, AX, CRO, CAZ, S, E, SPT, DO, AMC, CXM,CN, AK, K, CT | 1 | 14/19 | 7/10 | 0.7 |
| XL | P, AX, CRO, CAZ, S, E, SPT, DO, AMC, CXM, CTX, CN, K, CT | 1 | 14/19 | 7/10 | 0.7 |
| XLI | P, AX, CRO, CAZ, S, E, SPT, DO, AMC, CXM, CTX, CN, AK, K, APR | 1 | 15/19 | 6/10 | 0.8 |
| XLII | P, AX, CRO, CAZ, S, E, SPT, DO, AMC, CXM, CTX, CN, AK, K, CT | 1 | 15/19 | 7/10 | 0.8 |
